# Identification of recurrent focal copy number variations and their putative targeted driver genes in ovarian cancer

**DOI:** 10.1186/s12859-016-1085-7

**Published:** 2016-05-26

**Authors:** Liangcai Zhang, Ying Yuan, Karen H. Lu, Li Zhang

**Affiliations:** Department of Bioinformatics and Computational Biology, The University of Texas MD Anderson Cancer Center, 1400 Pressler St, Unit 1410, Houston, TX 77401 USA; Department of Biostatistics, The University of Texas MD Anderson Cancer Center, 1400 Pressler St, Unit 1410, Houston, TX 77401 USA; Department of Gynecologic Oncology and Reproductive Medicine, The University of Texas MD Anderson Cancer Center, Houston, TX USA; Department of Statistics, Rice University, Houston, TX USA; Department of Biophysics, College of Bioinformatics Sciences and Technology, Harbin Medical University, Harbin, China

**Keywords:** Copy number variations, Oncogenes and tumor suppressor genes, Focal and broad CNV, Cancer

## Abstract

**Background:**

Genomic regions with recurrent DNA copy number variations (CNVs) are generally believed to encode oncogenes and tumor suppressor genes (TSGs) that drive cancer growth. However, it remains a challenge to delineate the key cancer driver genes from the regions encoding a large number of genes.

**Results:**

In this study, we developed a new approach to CNV analysis based on spectral decomposition of CNV profiles into focal CNVs and broad CNVs. We performed an analysis of CNV data of 587 serous ovarian cancer samples on multiple platforms. We identified a number of novel focal regions, such as focal gain of ESR1, focal loss of LSAMP, prognostic site at 3q26.2 and losses of sub-telomere regions in multiple chromosomes. Furthermore, we performed network modularity analysis to examine the relationships among genes encoded in the focal CNV regions. Our results also showed that the recurrent focal gains were significantly associated with the known oncogenes and recurrent losses associated with TSGs and the CNVs had a greater effect on the mRNA expression of the driver genes than that of the non-driver genes.

**Conclusions:**

Our results demonstrate that spectral decomposition of CNV profiles offers a new way of understanding the role of CNVs in cancer.

**Electronic supplementary material:**

The online version of this article (doi:10.1186/s12859-016-1085-7) contains supplementary material, which is available to authorized users.

## Background

DNA aberrations in cancer can take many different forms, ranging from mutations, translocations, inversions and copy number variations (CNV) [1, 2]. The scope of the aberrations can range from single nucleotides to whole chromosomes. Recent analyses of CNVs in various types of cancers showed that their scope primarily exhibits two modes: it is either focal, which is limited to a small fraction of a chromosome, or very broad, which extends to a large fraction of a chromosome arm [3, 4]. Mechanistically, it was found that the focal CNVs (*f*CNVs) occur due to errors in DNA repair and the broad CNVs (*b*CNV) occur due to incorrect segregation of chromosomes during mitosis [1, 5, 6]. Regions of frequent *f*CNV are particularly important in cancer studies because they are believed to encode key genes driving cancer growth [7]. Many of the known oncogenes, such as ERBB2, EGFR and CCND1, are frequently amplified [8–12] and many of the known tumor suppressor genes (TSGs), such as CDKN2A, PTEN, NF1 and RB1, are frequently depleted in various types of cancers [13–15]. Thus, it is generally believed that recurrent focal gains are associated with oncogenes_ENREF_3 and focal losses associated with TSGs [16].

However, it remains to be a challenge to delineate the targeted oncogenes and TSGs from the recurrent CNVs [17–19]. For example, from an analysis of over 3000 cancer genomes, Beroukhim et al. identified 150 focal regions [4] that were supposed to be the hotspots of cancer driver genes, but only less than 25 % of the regions contained known oncogenes or TSGs. To explain the phenomena, it was proposed that some CNVs may rise from inherently fragile sites and gene poor regions. Solimini et al. proposed a ‘gene island’ theory [16]: genes that stimulate/inhibit tumor growth may distribute very unevenly across the genome. Such genes are not classical oncogenes or TSGs as they only have minor effects on tumor growth individually. However, the minor effects collectively can make a big difference through evolution of the cancer cells and produce the patterns of frequent gains/losses as we have observed. Sharon J. Diskin et al. [20] developed a statistical model called Significance Testing for Aberrant Copy number (STAC) to evaluate the randomness of the distribution of CNVs in tumors. STAC uses *p*-values to prioritize regions for down-stream analysis.

To better understand how CNVs are related to cancer driver genes, we analyzed CNVs in serous ovarian cancer, which is a type of cancer that contains relatively more CNVs than others. The dataset contained samples collected from 587 patients assayed on three different microarray platforms (Additional file 1: Table S1). Both primary tumors and adjacent normal tissues were assayed. An analysis of the dataset was reported previously by the TCGA consortium [21]. A statistical algorithm called GISTIC was used in several studies [3, 22, 23] to identify the regions that were highly enriched with copy number gains/losses. Several well-known cancer driver genes, such as RB1, PTEN and NF1, were suggested to be the targets of the regions. However, the targeted cancer genes of 70 (62 %) of the 113 regions are not known. The results reported by the TCGA consortium were largely consistent with earlier analyses of ovarian cancer, which used smaller sample sizes and obtained CNVs at lower resolution [24–29].

**Fig. 1:**
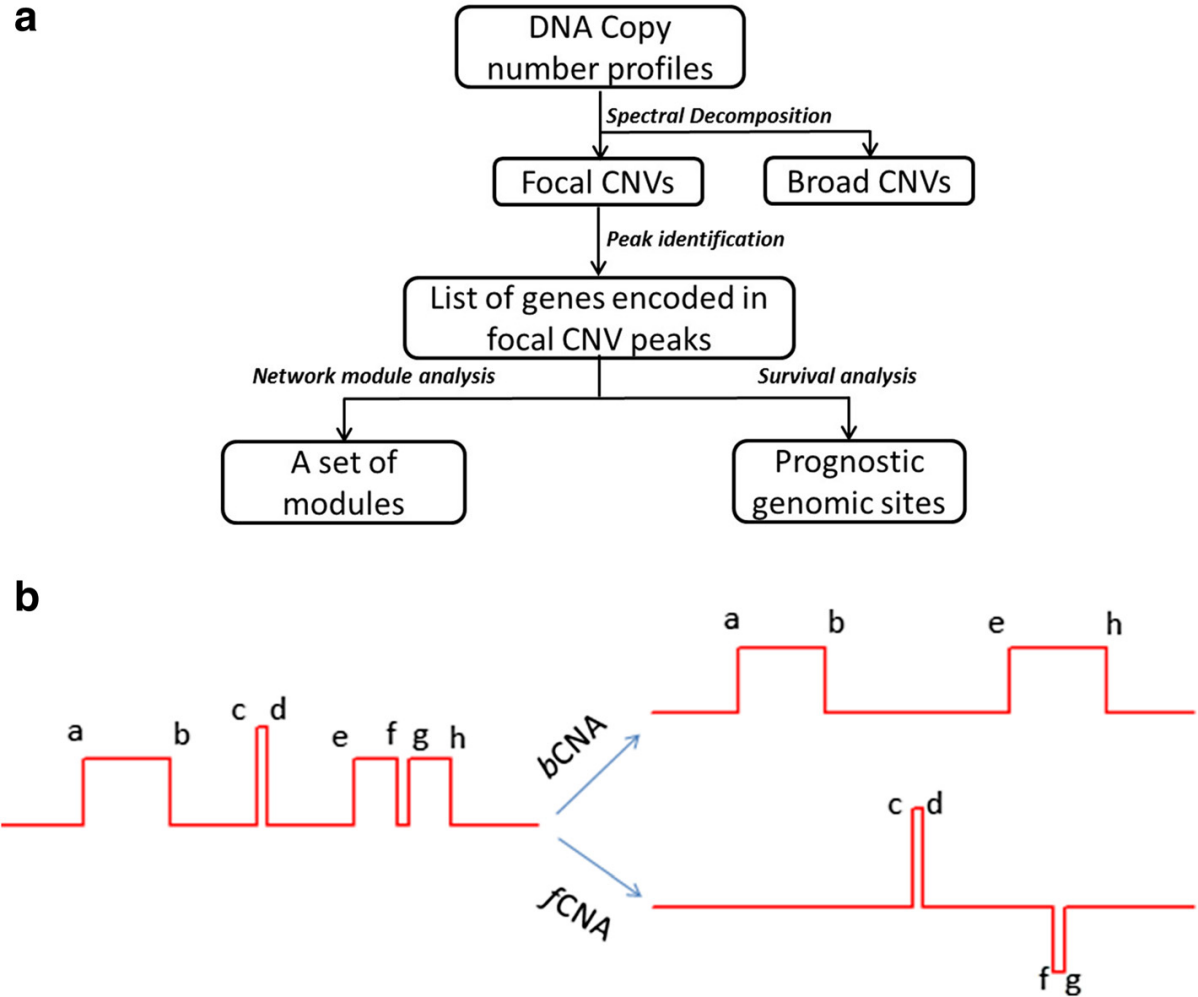
Analysis framework. **a** The flowchart of our analysis. **b** Schematic illustration of spectral decomposition of CNV profile. The red line on the left shows a CNV profile with vertical position representing the copy number and horizontal position representing chromosomal location. The letters in the figure mark the change points. The red lines on the left show the decomposed profiles, the *b*CNV on the top, and *f*CNV on the bottom. The segments $\overline{ab}$ and $\overline{eh}$ are considered broad gains. $\overline{cd}$ is a focal gain and $\overline{fg}$ is focal loss

We developed a new method aimed at identifying the focal CNVs that drive cancer progression (See in Fig. 1a). The method is designed to decompose a CNV profile into a focal profile and a broad profile based on the hypothesis that a genomic region may undergo multiple modes of aberrations during cancer progression. We viewed a CNV profile as a spectrum, in which the high frequency component corresponded to *f*CNVs and the low frequency component corresponded to *b*CNVs. Figure 1b illustrates the method schematically. To obtain the *b*CNV profile, we used the running median smoothing algorithm, which was originally proposed by Tukey [30]_ENREF_2 to smoothen time series data that follow a piecewise-constant model. The algorithm works by taking the median value in a scanning window over the CNV profile (See details in method section). The focal profile is computed from the difference between the CNV profile and the *b*CNV profile. This method is appealing because it preserves the shape of abrupt change points and it is robust for data that follow piecewise constant model. As shown in Fig. 1, the magnitude of broad CNV segment $\overline{eh}$ is not affected by focal CNV segment $\overline{fg}$ as long as $\overline{fg}$ span less than the half size of the scanning window. Using Fourier or wavelet transformations, it is also possible to separate high frequency from low frequency components from a CNV profile [31–35], but those transformations are not robust, nor do they preserve the shape of abrupt change points.

## Methods

To identify focal copy number variations and their putative cancer genes, our method consists of the following parts, which elaborate detailed steps of data collection from the TCGA data portal, CNV probe-level data de-noising and decomposition, identification of focal gains and losses, identification of peak regions and further downstream functional analysis (Network module and survival analysis).

The computational time for the CNV decomposition process is about 10–12 h on the high performance cluster (six nodes with 24 cores per node, Linux operating system); while for the rest procedures the time is less than 1 h. The proposed algorithm can be easily achieved using the detailed steps in method section, and the source R code is available at https://drive.google.com/drive/folders/0B6Q6G-z3ELntWllEd29IOVpyYzA.

### Data source

Copy number and mRNA expression data were downloaded from TCGA Data Portal (https://tcga-data.nci.nih.gov/tcga/dataAccessMatrix.htm) before September, 2012. Sample information of the 587 patients, progression free survival data, were summerized in Additional file 1: Table S1. Version hg18, Human Build 36.1 were used for annotating the genomic coordinates.

### CNV data processing

Let *x* be the vector of logarithm-transformed (*base* = 2) probe signals ordered according to the chromosomal positions in a sample. *x* is called a copy number profile. First, the profile is normalized using

1 $$ {x}_{normalized}=x- mode(x) $$

where *mode*(*x*) is mode value of *x*, *i.e.*, the peak position of density distribution funciton of *x*.

Second, the profile is smoothed to reduce noise,

2 $$ {x}_{smoothed}= runmed\left({x}_{normalized},w\right) $$

where *runmed* is the running median smoothing function with a scanning window of *w*, which was chosen to be 51, 51, 99 on the Agilent array, Illumina array, and Affymetrix array, respectively. The total number of probes are 9.6 × 10^5^, 1.19 × 10^6^, 1.87 × 10^6^, for the Agilent, Illumina, and Affymetrix arrays, respectively. Consequently, the chosen window sizes corresponded to approximately the same size on the chromosomes, circa 120 kb. The smoothing algorithm treated CNV events that spaned less than half of the window size as noise and ignored them. Thus, gain/loss fragments that were smaller than 59 kb were omitted in the subseqeunt analysis.

Third, because there is little change in the smoothed profile between neighboring probed sites, we chose to represent the smoothed profile at a lower resolution to reduce data dimension, *i.e.*,

3 $$ {x}_{reduced}={x}_{smoothed}\left[1,\ 1+w/3,1+2w/3,\dots,\ n\right] $$

where *n* is length of *x*_*smoothed*_.

To obtain the broad CNV profile and focal CNV profile, we used

4 $$ {x}_{bcnv}= runmed\left({x}_{reduced},wb\right) $$

5 $$ {x}_{fcnv}={x}_{reduced}-{x}_{bcnv} $$

where *wb* was chosen to be 641, 641, 793 on the Agilent, Illumina, and Affymetrix arrays, respectively. The window sizes correspond to approxmiately 32 Mb on the chromosomes, which means that CNV segments longer than 16 Mb are treated as broad changes and CNV segments shorter than 16 Mb are treated as focal changes. The results obtained in this study were not very sensitive to the choice of *wb*. Additional file 2: Figure S1 showed that the results were similar for *wb* between 30 and 40 Mb.

### Identification of focal gains or losses

At any genomic locus, the copy number has three states: gain, neutral, loss, which was determined as follows:

6 $$ {x}_{fcnv}\left\{\begin{array}{c}\hfill >\kern0.75em 3\varepsilon :\kern9em  gain;\hfill \\ {}\hfill \kern0.5em >-3\varepsilon \kern0.5em  and < 3\varepsilon :\kern2.5em  neutral;\hfill \\ {}\hfill < -3\varepsilon :\kern9.5em  loss\hfill \end{array}\right. $$

where *ε* is the estimated error of *x*_*fcnv*_.

### Identification of peak positions and their confidence intervals

To identify peak positions of focal CNV distribution, we used a scanning window of 41 *x*_*fcnv*_ sites to search for local maximums. Peaks with maximum value less than 8 were ignored.

To estimate the confidence intervals of the peak positions, a bootstrapping method [36, 37] was used. Boostrap samples were constructed using random sampling with replacement from the 587 focal CNV profiles. 500 sets of bootstrap samples were created, each set containing 587 profiles. For each set of the profiles, peak positions were identified. Because the number of such peak positions from bootstrap samples may be different from number of the original peaks, it is not possible to pair-up the two kinds of peaks one-to-one. To identify a new peak position for each orginal peak, we used the nearest peak position in the bootstrapping set with regard to each original peak to represent bootstrapped peak position. From the 500 sets of bootstrap samples, 500 sets of peak positions were obtained. The top 2.5 percentile and bottom 2.5 percentile of the bootstrapped peak positions were used as the estimates of 95 % confidence interval of each original peak.

### Identification of copy number polymorphism (CNP) from normal tissues

To identify CNPs that rise from germline mutation, we used focal CNV profiles of adjacent normal tissue samples. Because some of the samples appeared to be outliers as they contained a large number of CNPs, we suspected that they have been contaminated by tumor cells or the data had poor quality and decided to exclude them. We computed the number of CNPs for each sample as number of sites with |*f*CNV| > 5***ε***, where $\boldsymbol{\varepsilon}$ is the estimated error. Five samples on the Agilent platform with largest number of CNPs were determined as outliers and were excluded. Similarly, 61 and 48 normal samples were excluded from Affymetrix and Illumina arrays, respectively. After the removal of outliers, 240, 512 and 466 samples were included for CNP identification on the Agilent, Illumina, and Affymetrix arrays, respectively.

### Putative cancer driver genes based on *f*CNV and literature search

We created a list of cancer related genes from combining the cancer genes lists in OMIM database (http://omim.org/), Cancer Gene database of MSKCC [38], Sanger cancer gene census [39], TAG (http://www.binfo.ncku.edu.tw/TAG/) and TSGene databases (http://bioinfo.mc.vanderbilt.edu/TSGene/). The list contains 11,595 gene symbols.

An overlapping gene set of 200 genes was obtained between focal region genes and this cancer gene list.

We found 1245 *mRNA* transcripts were located in the 42 focal regions that were identified from our analyses. 200 out of the 1245 were contained in the list of cancer related genes.

For each of the 200 genes, we examined the references listed on GeneCards (http://www.genecards.com). A gene was defined as an oncogene if it has been reported that over-expression or activation mutation of this gene can cause cancer, promote cancer growth or metastasis. A gene was defined as a TSG if knockdown or deleterious mutation of this gene can cause cancer, promote cancer growth or metastasis.

### Survival analysis

We used Cox proportional hazard model to search for genomic loci where *f*CNVs or *b*CNV are correlated with progression free survival. At each the *j*^th^ genomic site, we constructed two models:

7 $$ {M}_{f, bcnv}\left[j\right]= coxph\left( Surv\left( time,\  status\right)\sim {x}_{fcnv}\left[j\right]+{x}_{bcnv}\left[j\right]\right) $$

8 $$ {M}_{cnv}\left[j\right]\kern1em = coxph\left( Surv\left( time,\  status\right)\sim {x}_{cnv}\left[j\right]\right) $$

where *x*_*fcnv*_ is *f*CNV, and *x*_*bcnv*_ is the *b*CNV, and *x*_*cnv*_ = *x*_*fcnv*_ + *x*_*bcnv*_. *coxph* is an *R* function that constructs an object of the cox proportional model [40]. *Surv*() creates a survival data object with censored *time* and *status*. Model A used the decomposed CNV profiles. Model B used CNV profiles without the decomposition.

### Cox model for screening of prognostic sites based on mRNA Expression data

For each gene *i*, a single-variate Cox model is set to be:

$$ {M}_i = coxph\left( Surv\left( time,\  status\right) \sim {g}_i\right) $$

where *g*_*i*_ is the logarithm transformed mRNA expression levels of the gene and *coxph* is an *R* function to construct an object of the cox proportional survival model. *Surv*() creates a survival data object with censored *time* and *status*.

### Network/module analysis on genes in *f*CNV regions

We used the NetBox server (http://cbio.mskcc.org/netbox), which is pre-loaded with a Human Interaction Network (HIN) derived from curated literature. HIN contains 9264 nodes and 68111 edges. The genes in the focal regions identified from our analyses were mapped to HIN to identify the core modules. The parameters used in the NetBox analyses were as follows: shortest path threshold = 2; *p*-value threshold = 0.05; number of global trials = 1000; number of local trials = 100.

### Assessment of sensitivity and specificity based on computer simulated data

To generate simulated CNV profile, we conducted the simulation as follows: we set the dimension of a CNV profile having *L* = 1.6 × 10^4^ probes for *N* = 500 patients. The measurement error of the probe signals is set to follow normal distribution with a standard deviation *ε* = 1. Each patient has a normal CNV profile and a tumor CNV profile. A fraction of the patients (f*N) have an amplicon in their tumor CNV profile. The height (*h*) and the width (represented as number of probes *n*) of the amplicon are set to be variable. We then apply the decomposition algorithm to identify status of gain/loss at the center of the CNV profiles. False positive rates, true positive rates, true negative rates, and false negative rates were calculated according to nominal truth. The significance *p*-value is calculated based on the test of whether the number of focal gains is significantly greater than the number of focal losses in the tumors at a particular genomic locus.

## Results and discussion

Using the decomposition method, we performed the analysis of focal copy number identification for the ovarian cancer data from the TCGA data portal. We found focal regions contain putative cancer driver genes, and have significant co-occurrences with tumor suppressors and oncogenes. Also, we investigated the relationship between gene expression alterations and their copy number variations in focal copy number regions. Finally, we checked if focal copy number variations play a role in patients’ survival. Detailed results are elaborated in the following (or in Additional file 2: Supplementary information).

### Identification of focal regions containing putative cancer drivers

**Fig. 2:**
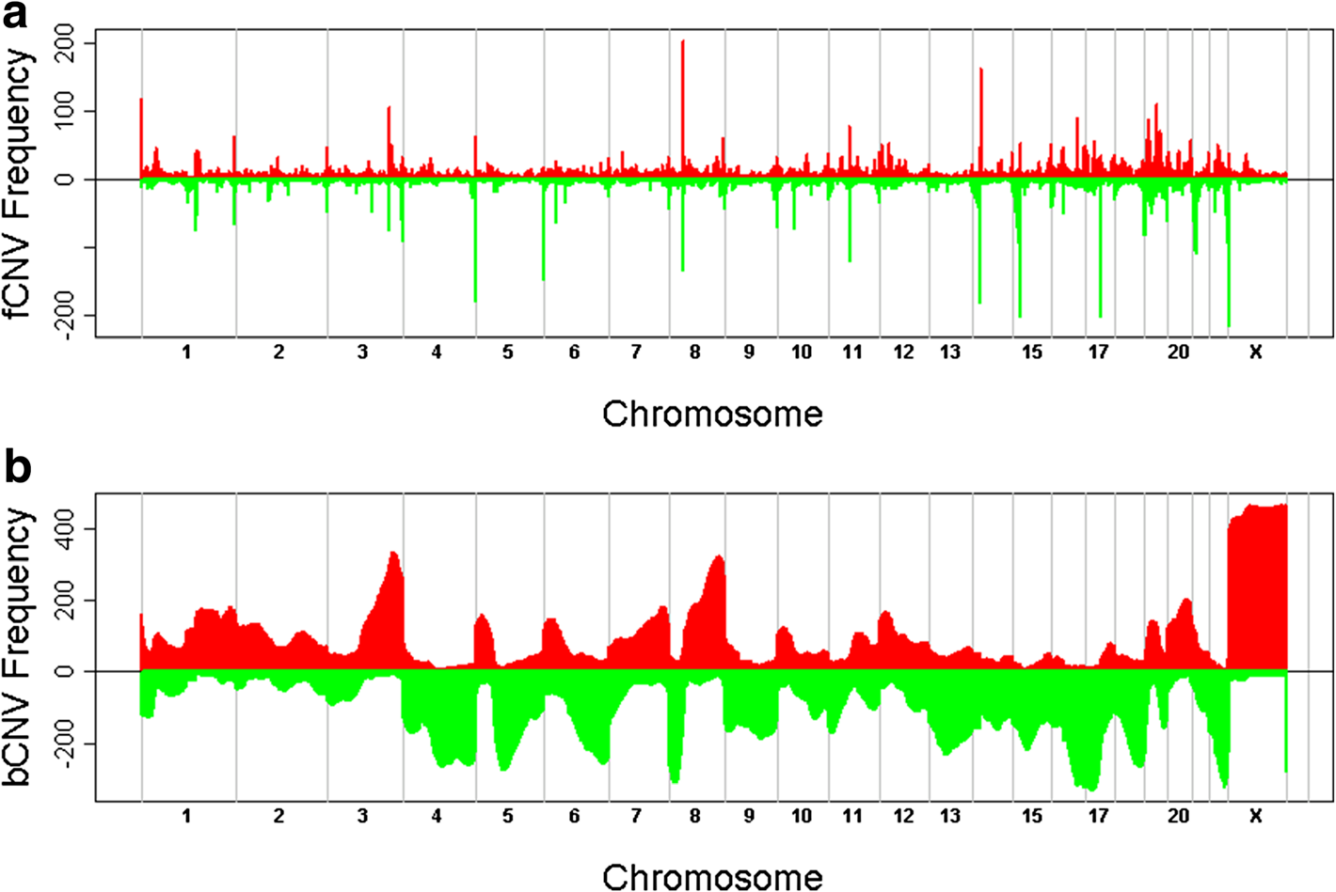
Distribution of focal and broad CNVs in 587 ovarian cancer samples. **a** *f*CNV; (**b**) *b*CNV. The *x*-axis shows the chromosomes. Data regarding the Y-chromosome were removed. The *y*-axis shows the numbers of samples with gains as positive numbers in red and numbers of samples with losses as negative numbers in green. Numerical data of the distributions can be found in Additional file 1: Table S2

Figure 1a shows a flowchart of our analysis process. First, we obtained the distributions of focal changes and broad changes across the human genome (Fig. 2 and Additional file 1: Table S2). We evaluated the distributions for tumor and normal tissue samples separately on each of the three microarray platforms (Additional file 2: Figure S2). Then we identified the peak positions of the *f*CNV distribution and calculated the confidence interval (CI) of the peak positions using bootstrap samples (See in methods). We searched for focal regions that met the following criteria: (1) Peak height ≥ 8; (2) The 95 % CI of the peak position is less than 1 million base pairs. (3) Less than 4 gains or losses were found in the normal tissue samples within the 95 % CI. This criterion filtered out regions that are polymorphic in the healthy population; (4) The number of focal gains must be significantly different from the number of losses in the same region.

We identified 42 focal regions that met our criteria, which are composed of 26 focal gain regions and 16 focal loss regions (Additional file 1: Table S3). These regions collectively encode 1245 transcripts (809 gene symbols). Based on literature search, we found 47 oncogenes in the focal gain regions and 15 TSGs in the focal loss regions, which we regarded as putative driver genes (Additional file 1: Table S3).

We compared results with previous analysis of ovarian cancer [21] using GISTIC, which found 50 focal regions with peak-width less than 1 Mb. 25 of the 50 had overlaps with the focal regions in our analysis. Additionally, we compared our results with a report of GISTIC analysis of multiple cancer types [41], which found 75 focal regions with peak-width less than 1 Mb, and 29 of them overlapped with our focal regions.

**Fig. 3:**
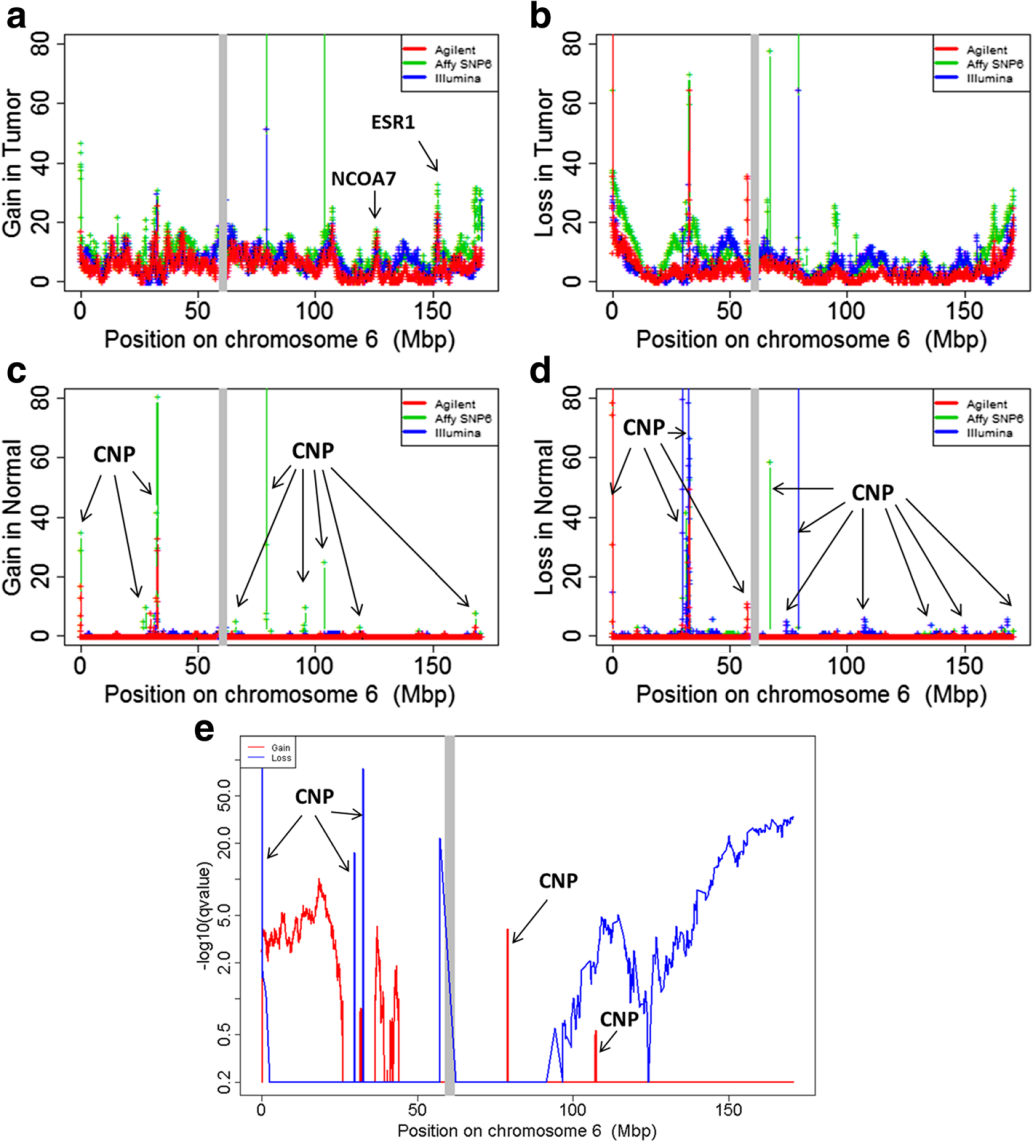
Focal gains and losses on chromosome 6. **a** Focal gains in tumor samples; (**b**) focal losses in tumor samples; (**c**) focal gains in normal samples, CNP means the copy number polymorphism in normal population; (**d**) focal losses in normal samples; (**e**) GISTIC results

We noticed a number of features that had not been reported in the previously. Our analyses unveiled recurrent focal gains in the midst of broad losses. For example, 6q loss is common in many cancers (Fig. 2b). Surprisingly, we found a focal gain region around 6q25.1. The focal gains in tumors were observed on all the three microarray platforms (Fig. 3a) but not in normal tissues (Fig. 3c, d). The region encodes an oncogene ESR1 known in many cancers including ovarian cancer [42–45]. The focal gain in the tumor samples was not identified using GISTIC 2.0 (Additional file 2: Figure S3), presumably because the gain of ESR1 can only be identified after CNV decomposition.

**Fig. 4:**
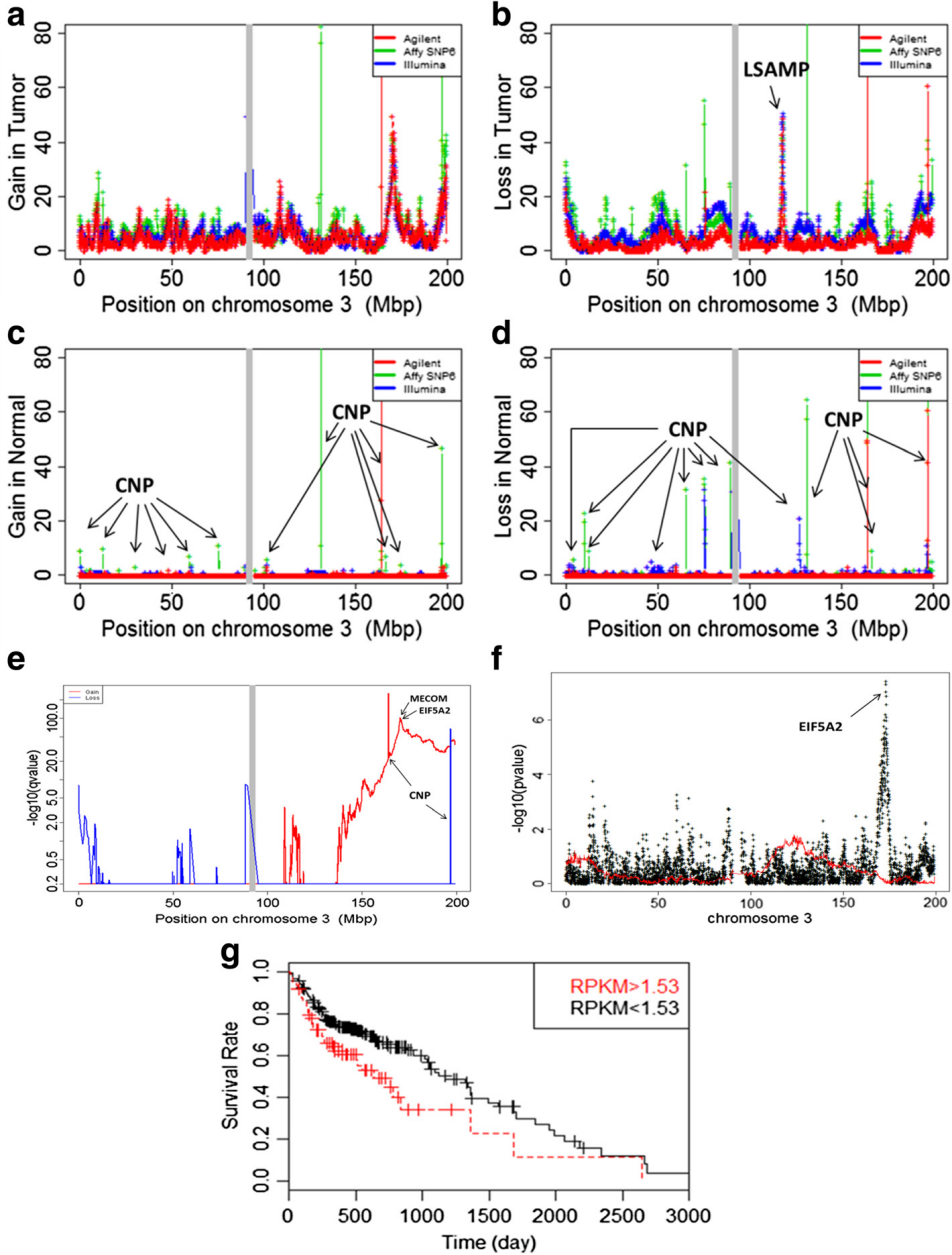
Focal gains and losses on chromosome 3. **a** focal gains in tumor samples; (**b**) focal losses in tumor samples; (**c**) focal gains in normal samples, CNP means the copy number polymorphism in normal population; (**d**) focal losses in normal samples; (**e**) GISTIC results; (**f**) PFS ~ *f*CNV + *b*CNV, the black points represent the prognostic power of *f*CNVs, while the red represent that of *b*CNVs; (**g**) Figure of KM-plot for EIF5A2. We used two Cox model analyses. Model 1: model: PFS ~ focal. Wald test *p*-value = 4.23E-08. Model 2: the samples were partitioned into two groups the upper quartile (RPKM > 1.53) and the rest (RPKM < 1.53). *P*-value = 0.006808, model: PFS ~ Group

Similarly, LSAMP located at 3q26 is an example of focal losses in the midst of broad gains (Figs. 2b and 4). The focal losses in tumors were observed on all the three microarray platforms (Fig. 4a) but not in normal tissues (Fig. 4c, d). LSAMP was identified as a candidate tumor suppressor and focal deletion LSAMP were reported in other cancers [46–50]. Expression of LSAMP was also shown to be associated with osteosarcoma progression [50]. GISTIC analysis of LSAMP region identified no significant deletions (Fig. 4e).

### Relationship between gains/losses and oncogenes/TSGs

**Fig. 5:**
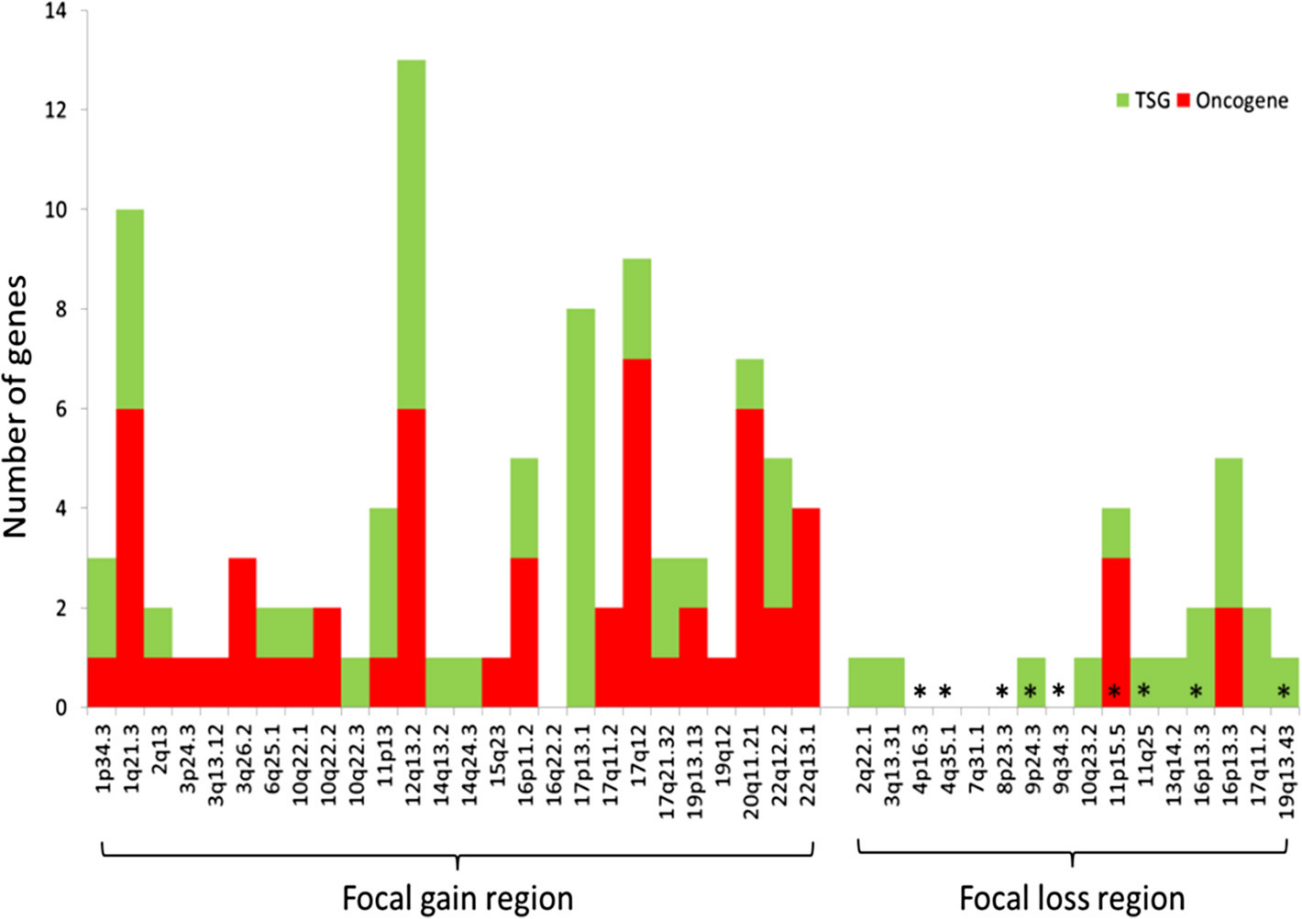
Known cancer genes (Oncogenes and TSGs) in regions of recurrent focal CNVs

Although it is widely believed that oncogenes are associated with the gains and TSGs associated with losses, the associations have not been evaluated quantitatively in previous studies. We have tested this association using our results. Figure 5 showed the numbers of focal regions that contained known cancer driver genes in the 42 focal regions identified in our analysis. Most of the regions contained oncogenes as well as TSGs. However, the oncogene-to-TSG ratio in the focal gain regions was different from that in the focal loss region: The ratio is 22:14 in the focal gain regions and 2:11 in the focal loss regions. The ratios differ by 8.6-fold, which is statistically significant (*p*-value = 8.30×10^−3^, Fisher’s exact test.)

Third, there were 9 regions of focal losses located at the ends of chromosomes (4p, 4q, 8p, 9p, 9q, 11p, 11q, 16p and 19p, in Fig. 5), which may be related to telomere loss in serous ovarian cancer [51–53]. In four of the focal regions, no known oncogenes or TSGs were found.

### Network modularity analysis of genes encoded in the focal regions

**Fig. 6:**
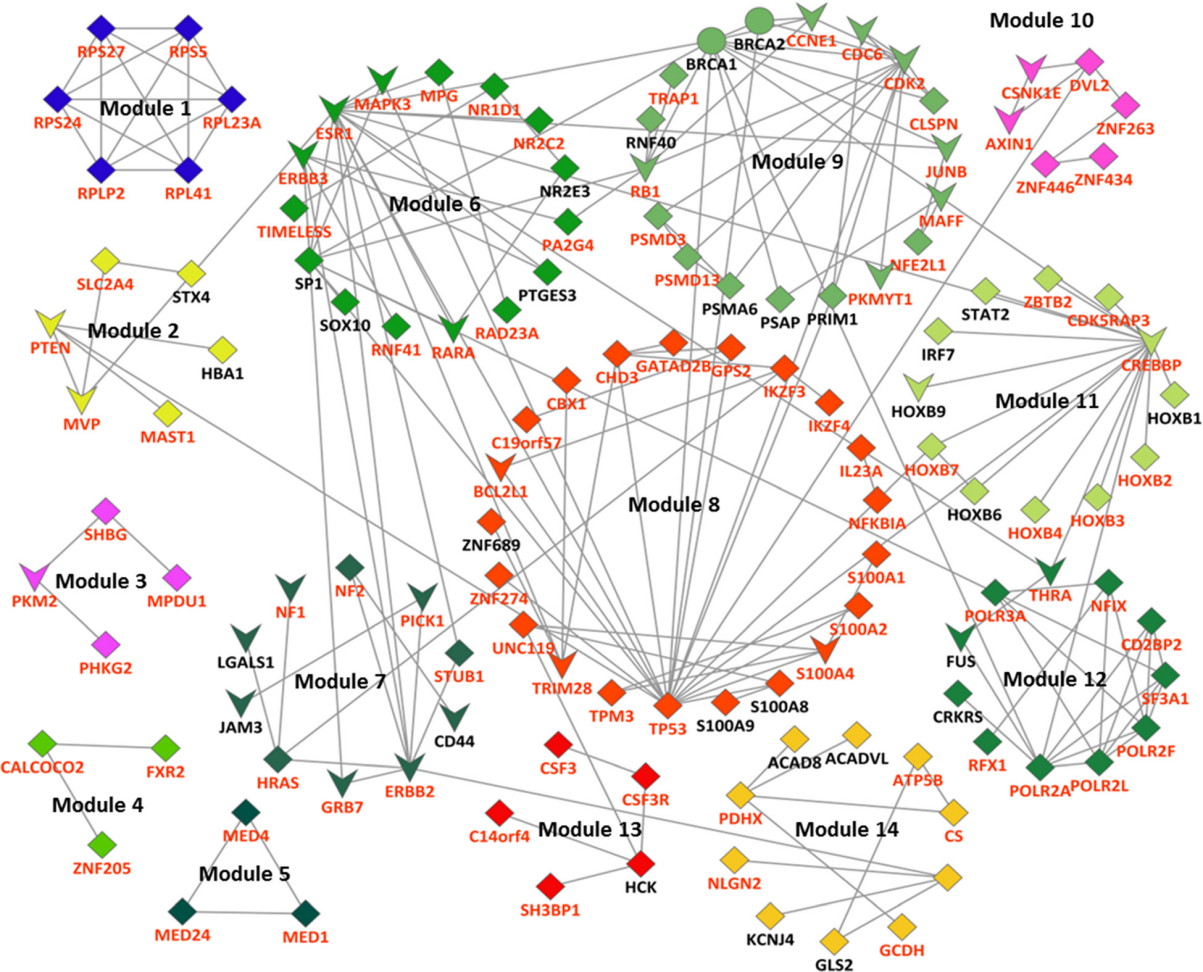
Network modules of cancer drivers in ovarian cancer. There are 130 nodes and 188 edges in this network. Nodes with the label in red have their *f*CNV changes responsive to gene expression. The ‘diamond/V’ node represents the gene is located within 42 focal regions. The ‘V’ node represents the current gene is a known-cancer driver based on our curated literature search

To explore the underlying relationship among the 1245 genes encoded in the 42 focal regions, we performed a network modularity analysis using NetBox [54]. 809 of 1245 transcripts can be mapped into the background network stored in NetBox. The NetBox analysis found a sub-network of 14 modules consists of 130 genes and 188 edges (Fig. 6). This sub-network has an overlap of 31 with the 62 putative drivers. This level of overlap is statistically significant (*p*-value = 6.45 × 10^−5^, Fisher’s exact test), which suggests that the driver genes are more “connected” than other genes.

### Association between focal CNVs and genes expression

**Fig. 7:**
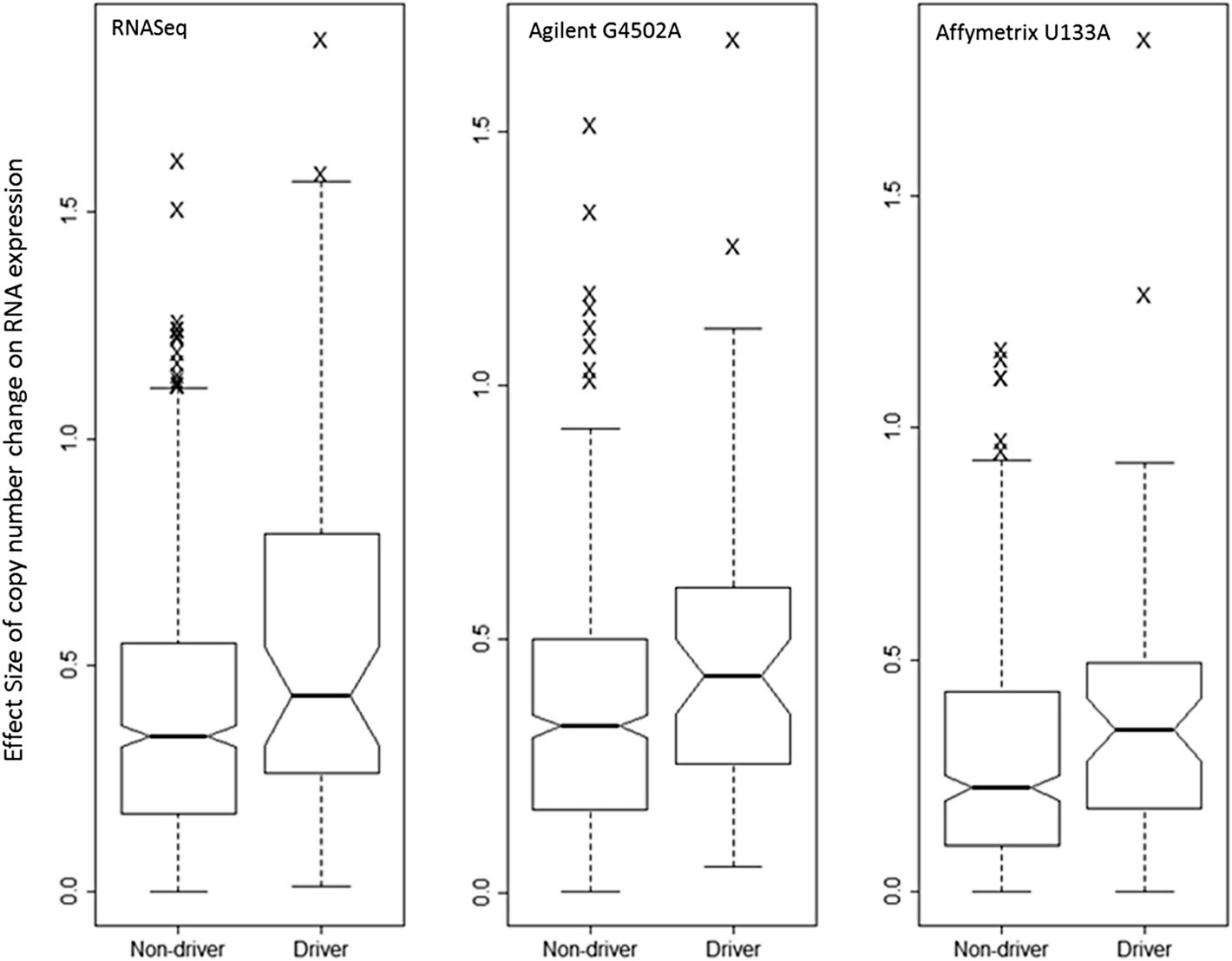
Effects of copy number change on RNA expression for cancer drivers and non-cancer drivers. The vertical axis shows effect size of copy number change on RNA expression as boxplots. In the focal gain regions, each data point used in the boxplots were computed from the average log-fold change of mRNA expression of a gene between tumor samples with focal gain and tumor samples without focal gain. In the focal loss regions, each data point used in the boxplots were computed from the average log-fold change of mRNA expression of a gene between tumor samples without focal loss and tumor samples with focal loss. The boxplots were generated from data on three different platforms: RNAseq, Agilent 244 K and Affymetrix U133A, corresponding to the panels on the left, middle and right, respectively. The differences in the effect sizes are significant (*p*-values =0.012, 0.0059, 0.020, for the left, middle and right panels, respectively)

To further explore the functional role of focal CNVs, we assessed association between focal CNVs and gene expression. We found that the focal changes have a greater effect on putative drivers (Fig. 7). Using gene expression data obtained on the RNAseq platform, the mean fold change in the gene expression in response to focal gain/loss is 0.54 for cancer driver genes and 0.39 for other genes, respectively. On the Agilent 244 K platform, the corresponding values were 0.48 and 0.36. And on the Affymetrix U133A platform, the values were 0.40 and 0.29.

### Association between focal CNVs and patient survival

We searched for genomic loci where CNVs were associated with patient survival. We used Cox proportional hazard model with the disease progression free survival (PFS) data of the patients. At each genomic site, we used *f*CNV and *b*CNV as two covariates to evaluate their association with PFS. We obtained similar results are similar on all of the three microarray platforms (Additional file 2 Figure S4 and Additional file 1: Table S4). The most significant loci for prognosis are listed in Table 1. Additional file 2: Figure S5 showed the *p*-values of the *f*CNV and *b*CNV in the Cox models. One of the most significant site was located at 3q26.2 (genome coordinate: Ch3:171–174 Mb). We found that mRNA expression of the genes encoded in the region is also significantly associated with PFS (Fig. 7). Interestingly, EIF5A2, which is one of the genes in the region, was found to be prognostic in ovarian cancer [55], colorectal carcinoma [56] and urothelial carcinoma [57]. Several other genes also had expression values significantly associated with PFS (*p*-value < 0.001, Wald test). These associations lend further support of the genomic with prognostic value.

**Table 1:** Prognostic loci of ovarian cancer progression. The (Wald test) *p* values were obtained from Cox proportional hazard model with *f*CNV and *b*CNV data on the Affymetrix array platform as covariates

Chr	Region(Mb)	*p*-value	Representative genes
1	239.017–239.540	2.7E-04	KMO
3	171.107–174.249	1.0E-05	ECT2, EIF5A2, CLDN11, MYNN, LRRC31, EVI1
4	19.677–20.283	8.2E-06	
12	22.873–23.388	6.5E-04	KIAA0528
18	24.633–24.729	1.7E-04	
X	32.237–32.418	5.8E-06	

### Test of *f*CNV detection algorithm with computer simulated data

To evaluate the performance of the algorithm that we developed for *f*CNV detection, we applied the algorithm into the computer-generated data. The simulated data were generated following a very simple scenario: The profile contains only one amplicon at the center (See Method section for details). Detection of the amplicon depends on the amplitude (height *h*) of the amplicon over the noise level (ε), and the width of the amplicon (*n*, number of probes). The false positive rate approaches to 0 when *h/ε* > 0.9 and *n* > 30 (Additional file 2: Figure S6.1 and S6.2). In addition, since one of the prerequisites of oncogenes is that the number of focal gains ought to be significantly greater than the number of focal losses, it follows that the frequency of patients with the amplicon cannot be too low for the positive/correct identification. We found that *f* should be greater than 0.02 such that we can have a *p*-value <0.05 when 500 patients were tested (Additional file 2: Figure S6.3). These results suggested that we may not be able to identify the cancer driver genes if the frequency is less than 2 % of the patients in our study, or the focal CNVs are too short (e.g., with fewer than 30 probes covering the aberrant genomic region).

## Conclusions

In this study, we developed a new approach to CNV analysis based on spectral decomposition CNV profile that separates focal CNV from broad CNVs. Using this approach, we performed an analysis of 587 serous ovarian cancer samples and found significant focal regions that are likely to contain cancer drivers. These regions have partial overlaps with regions that had been reported in previous analyses, but significant differences were also noted. Our results yielded a list of interesting findings, such as focal gains around ESR1, focal loss around LSAMP, prognostic site at 3q26.2 and sub-telomeric losses. 29 of the 42 focal regions from our analysis overlapped with the focal regions reported by previous pan-cancer analysis using GISTIC, which suggests that our results are in general agreements with previous analyses but also offered new focal regions of interest, which demand further investigations.

We also formerly tested the association between gain/loss and oncogene/TSG. Our results confirmed that the recurrent focal gains were significantly associated with the known oncogenes and recurrent losses associated with TSGs and the CNVs had a greater effect on the mRNA expression of the driver genes than that of the non-driver genes. Our results also showed focal CNVs had greater effects on expression of cancer driver genes than that of the non-driver genes. Our study demonstrated that spectral decomposition of CNVs offers a powerful new way of understanding the role of CNVs in cancer.

## Abbreviations

Affymetrix SNP 6.0, affymetrix genome-wide human SNP array 6.0; Affymetrix U133A, affymetrix human genome HTS U133A 2.0; Agilent 244 K, agilent 244 K custom human gene expression G4502A-07–3; Agilent G4447A 1 M, agilent SurePrint G3 human CGH microarray kit 1×1M; bCNV, broad copy number variation; CI, confidence interval; CNP, copy number polymorphism; fCNV, focal copy number variation; GISTIC, genomic identification of significant targets in cancer; Illumima 1 M-Duo, Illumina Human1M-Duo BeadChip; Mb, million base pair; PFS, progression free survival; RNAseq, whole transcriptome shotgun sequencing; RPKM, reads per kilobase per million of mapped reads; SNP, single nucleotide polymorphism; TCGA, the cancer genome atlas; TSG, tumor suppressor gene.

## Additional files

Additional file 1: Table S1. Sample information of 587 ovarian patients and normal tissue samples with Copy number variation and mRNA data on different platforms. **Table S2.** Distributions of focal gains/losses in primary tumors and adjacent normal tissue separately on each of the three microarray platforms. **Table S3.** List of genes encoded in the recurrent focal event regions. **Table S4.** Prognostic results of ovarian cancer progression using *f*CNV on three platforms. (XLSX 20403 kb) Additional file 2: The additional file contains all the details of additional/supplementary figures (**Figure S1**–**S6**) and tables (**Table S1**–**S4**). (DOCX 1574 kb)
