# Supplementary material for: Identification of recurrent focal copy number variations and their putative targeted driver genes in ovarian cancer
Source: BMC Bioinformatics. 2016 May 26;17:222. doi: 10.1186/s12859-016-1085-7 (PMC4881176; doi:10.1186/s12859-016-1085-7)
Supplement: Additional file 2: — The additional file contains all the details of additional/supplementary figures (Figure S1–S6) and tables (Table S1–S4). (DOCX 1574 kb) [file 12859_2016_1085_MOESM2_ESM.docx]

SUPPLEMENTARY INFORMATION

Table of Contents

[Supplementary figures and tables 2](#_Toc445063369)

[Figure S1 Evaluation on effects of different window sizes 2](#_Toc445063370)

[Figure S2 Distributions of focal gains/losses. 3](#_Toc445063371)

[Figure S3 Results of GISTIC analysis. 16](#_Toc445063372)

[Figure S4 Significance of association between CNV and PFS 30](#_Toc445063373)

[Figure S5 Significance of association between gene expression and PFS. 33](#_Toc445063374)

[Figure S6 Simulation results of the sensitivity and specificity for our decomposition method 35](#_Toc445063375)

[Table S1 Sample information 38](#_Toc445063376)

[Table S2 Distributions of focal gains/losses 40](#_Toc445063377)

[Table S3 List of genes encoded in the recurrent focal event regions. 43](#_Toc445063378)

[Table S4 Prognostic results of ovarian cancer progression using fCNV on three platforms. 44](#_Toc445063379)

[Abbreviations 46](#_Toc445063380)

# Supplementary figures and tables

## Figure S1 Evaluation on effects of different window sizes


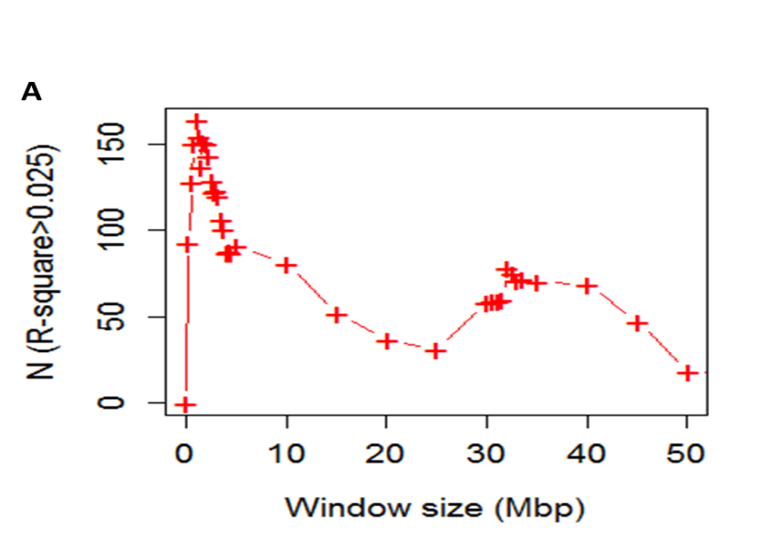


**Figure S1 Evaluation on effects of different window sizes.** *Y*-axia represents the number of probes (Cox survival model, R-square>0.025) and *x*-axis is scanning window size (Mbp).

## Figure S2 Distributions of focal gains/losses.

Figure S2 showed the frequency distributions on individual chromosomes of $f\mathrm{CNV}$ events in tumor and normal samples. Lines in red, green and blue represent data from Agilent, Affymetrix SNP6 and Illumina platforms, respectively. The $x$-axis represents physical position on a specific chromosome, and the $y$-axis represents the frequency of copy number gains or losses.

*Chromosome 1*


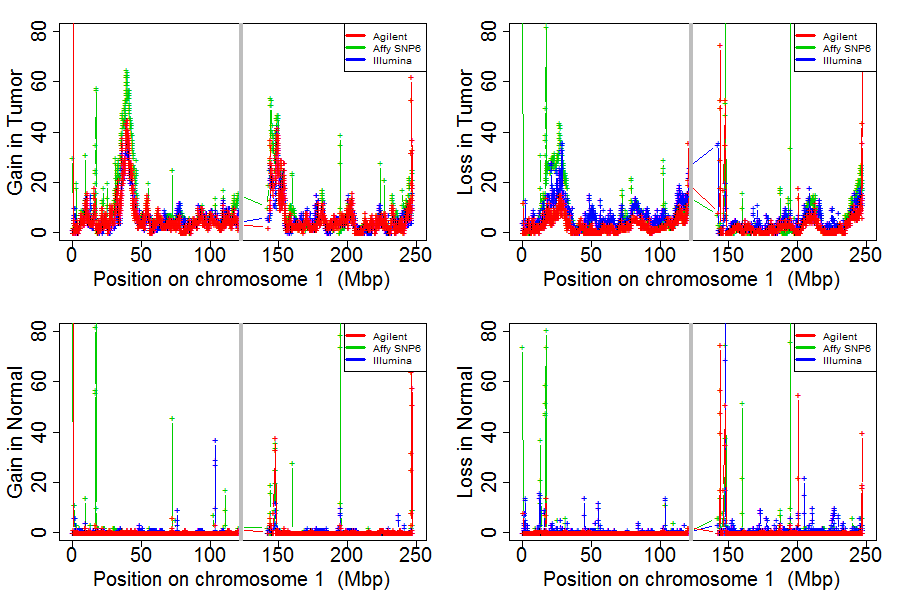


*Chromosome 2*


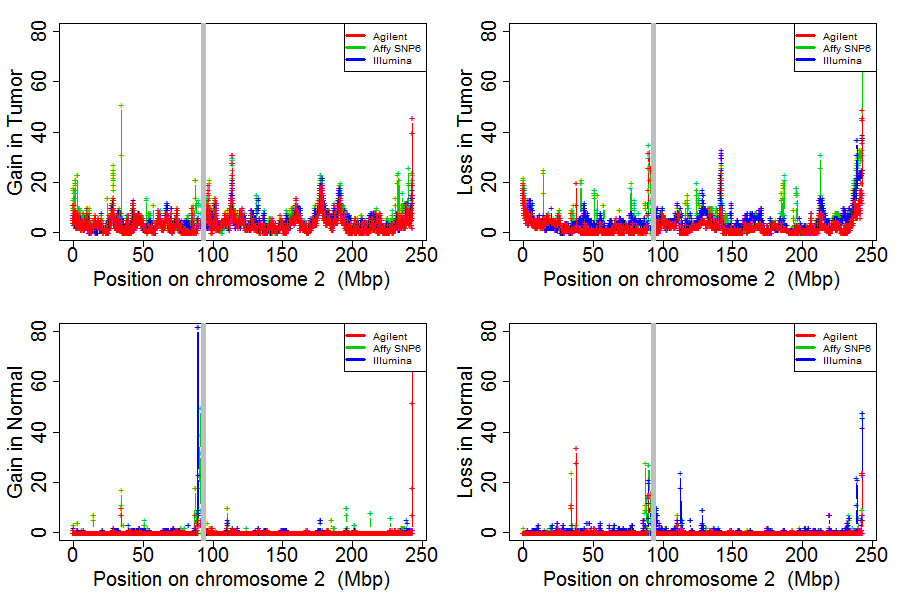


*Chromosome 3*


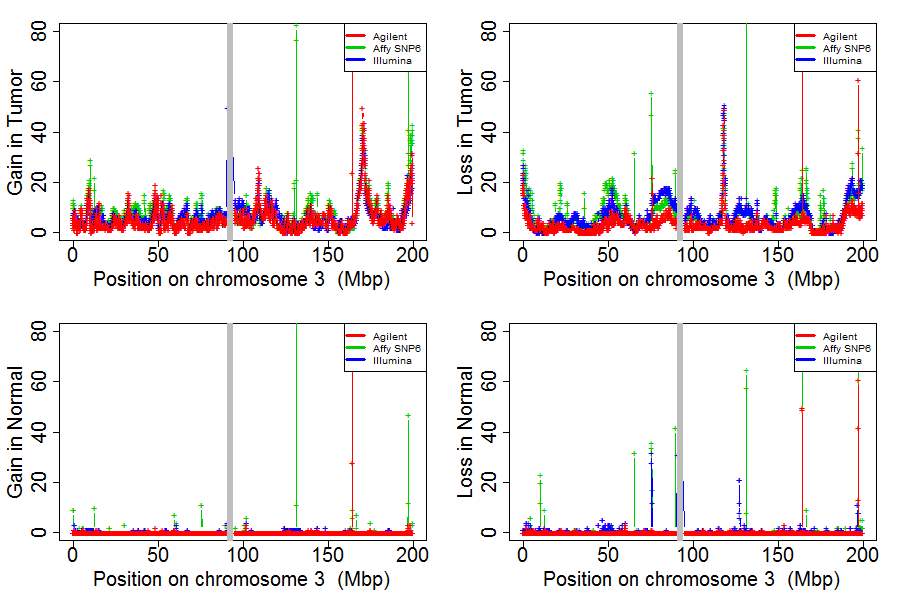


*Chromosome 4*


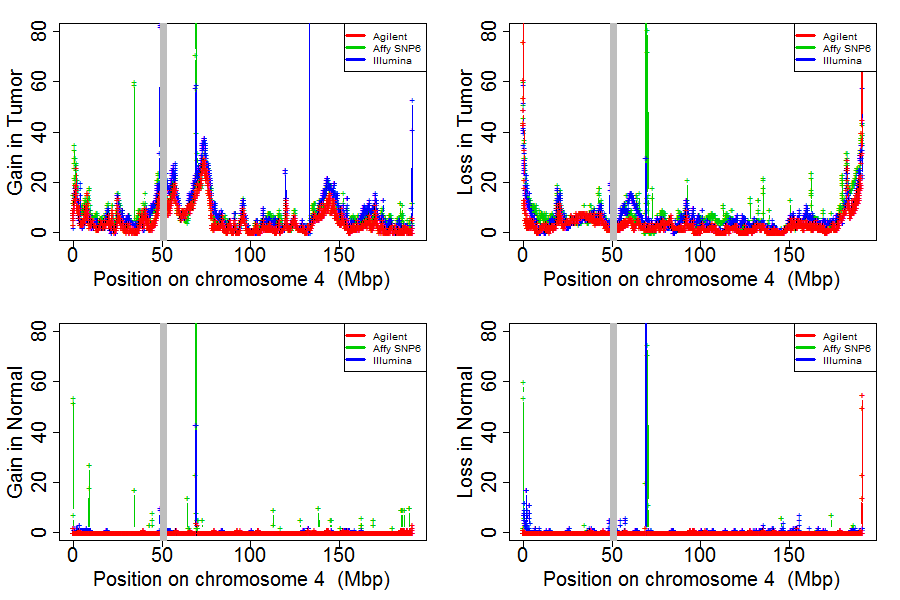


*Chromosome 5*


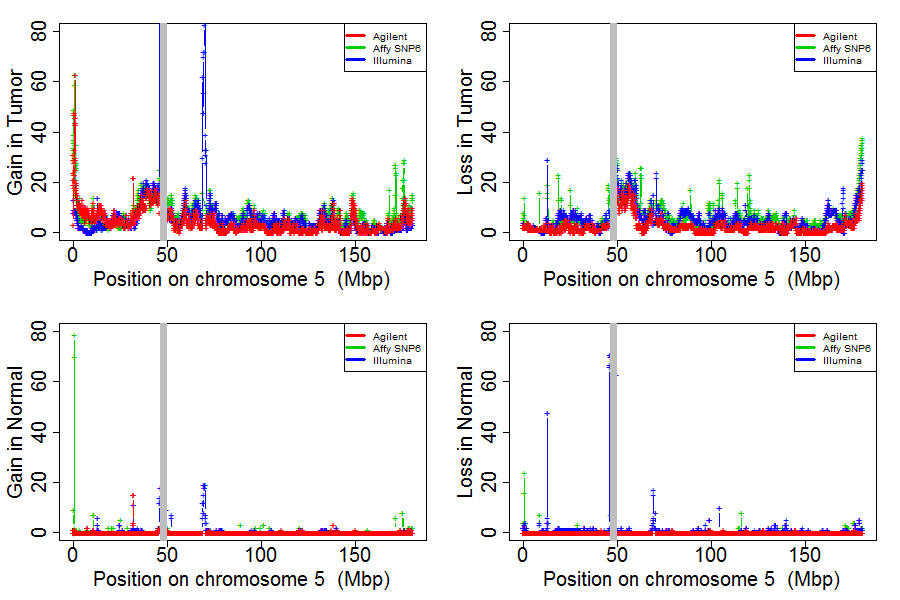


*Chromosome 6*


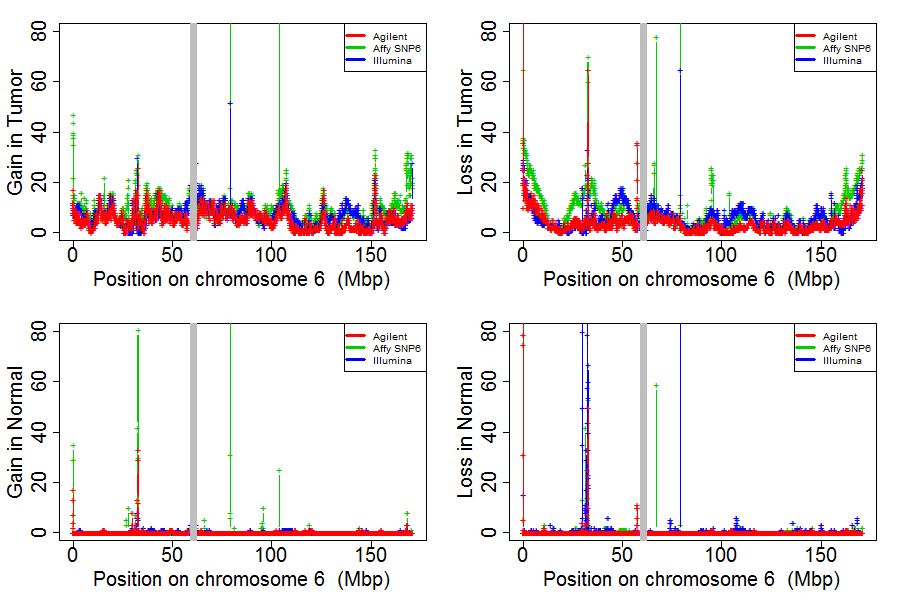


*Chromosome 7*


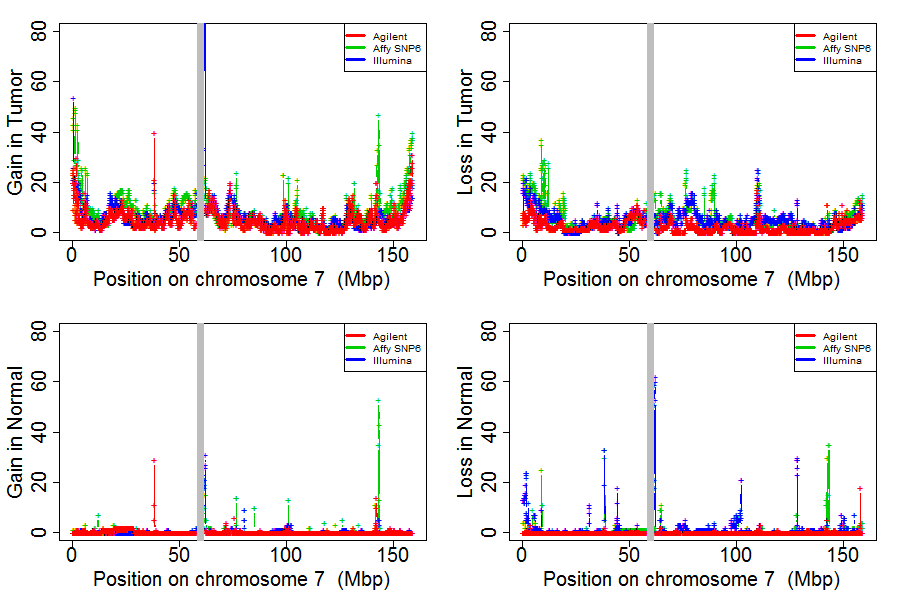


*Chromosome 8*


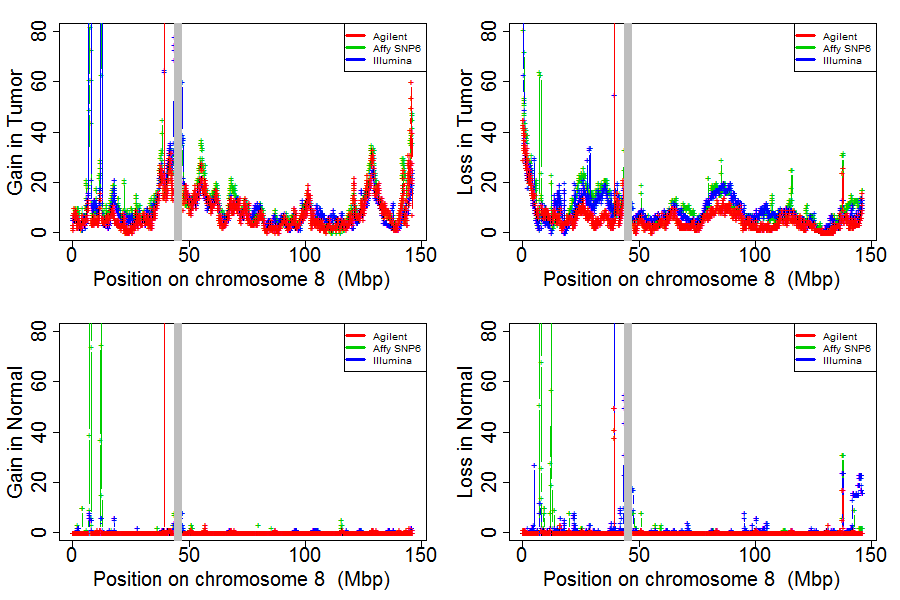


*Chromosome 9*


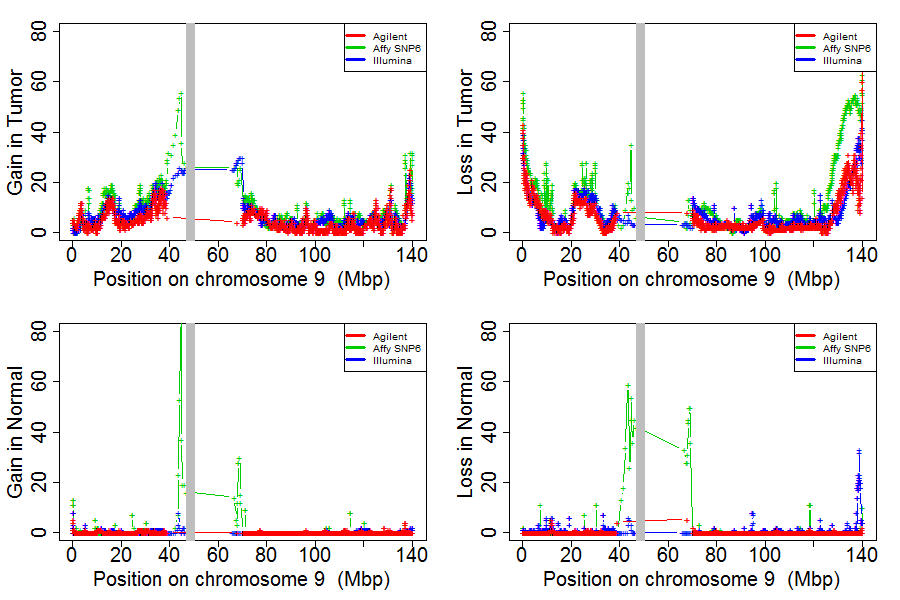


*Chromosome 10*


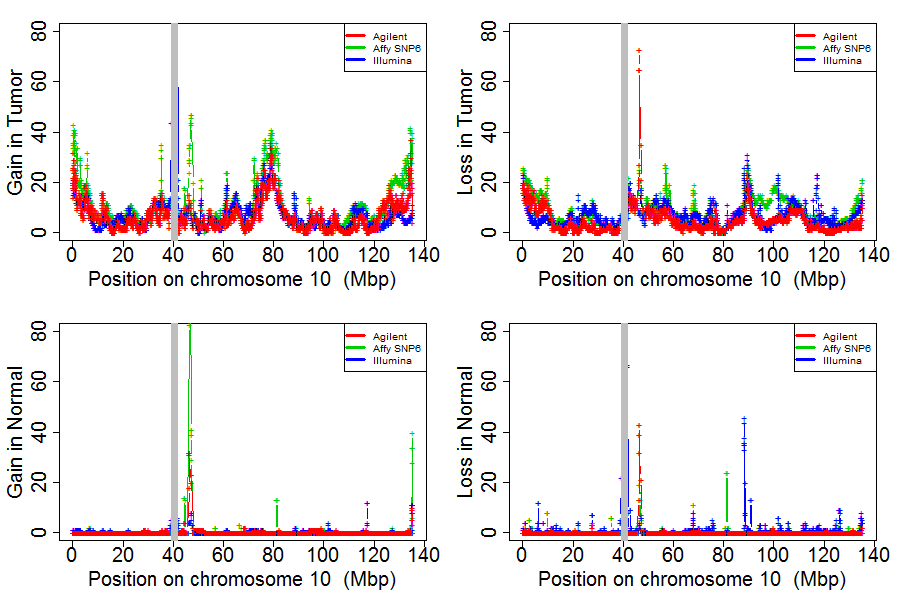


*Chromosome 11*


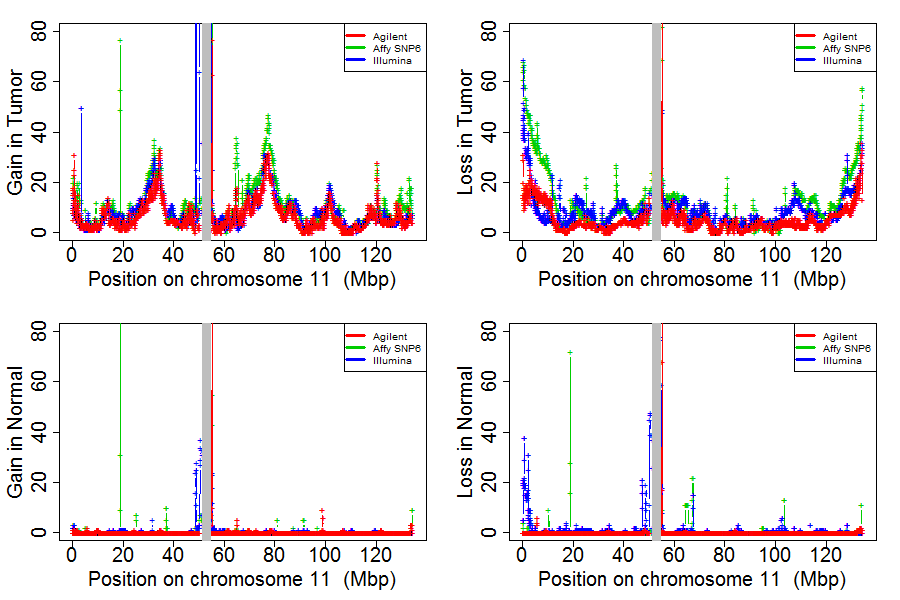


*Chromosome 12*


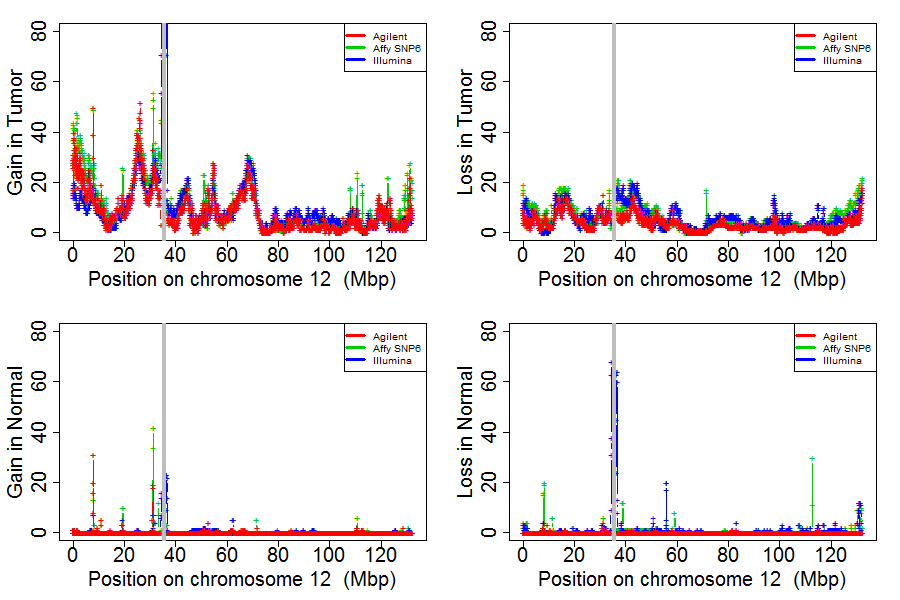


*Chromosome 13*


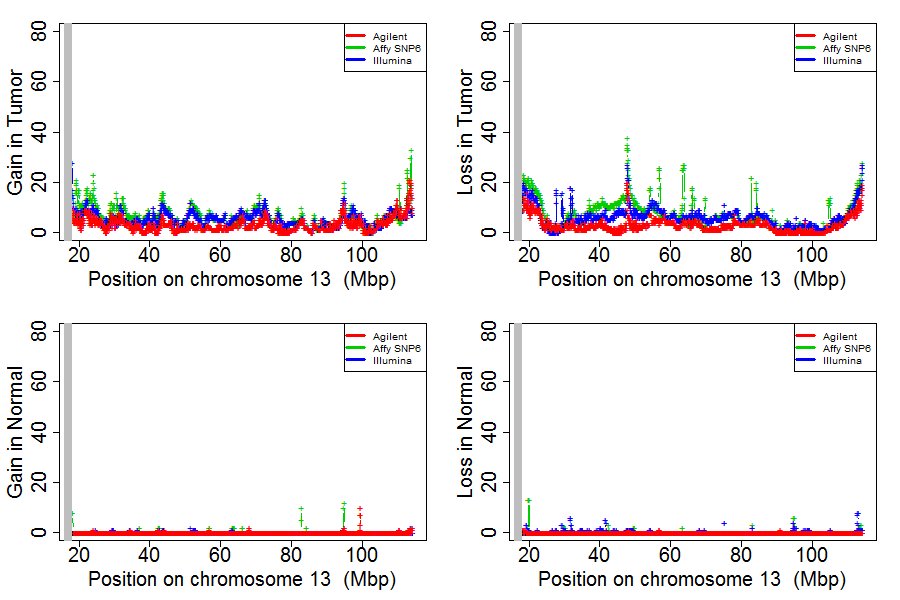


*Chromosome 14*


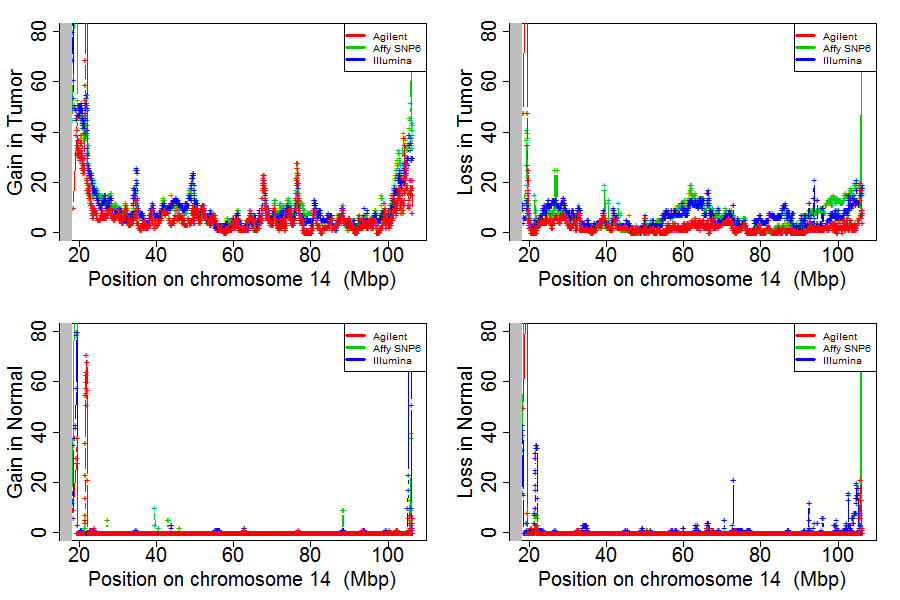


*Chromosome 15*


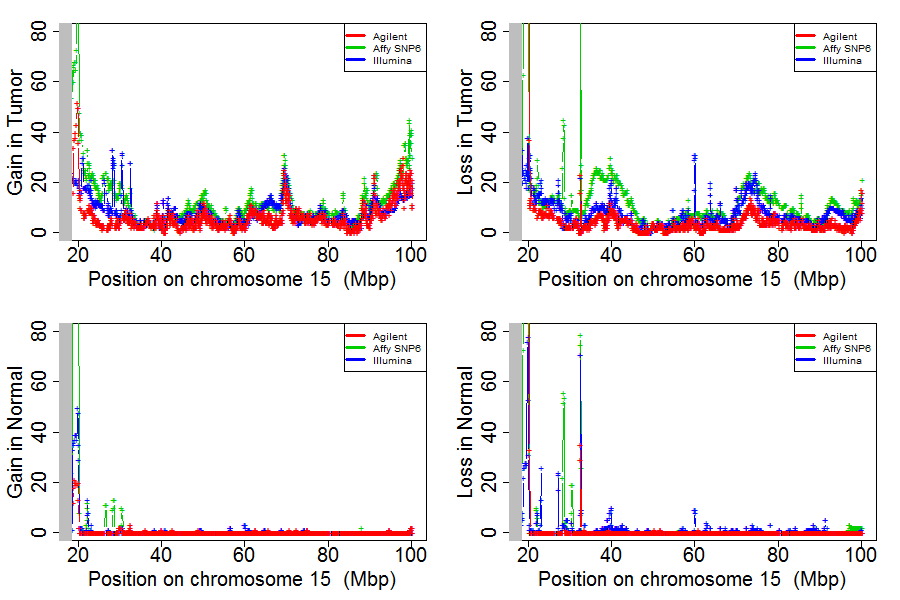


*Chromosome 16*


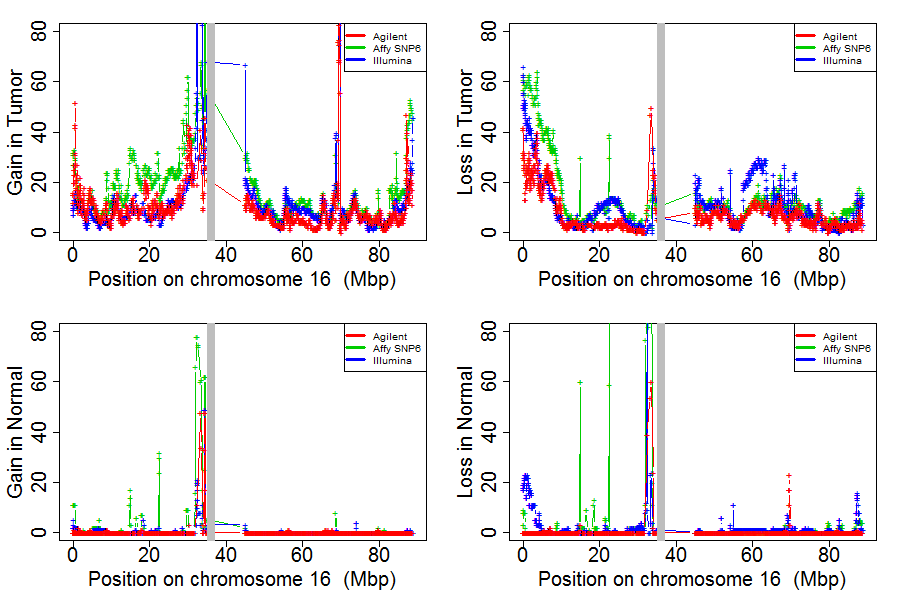


*Chromosome 17*


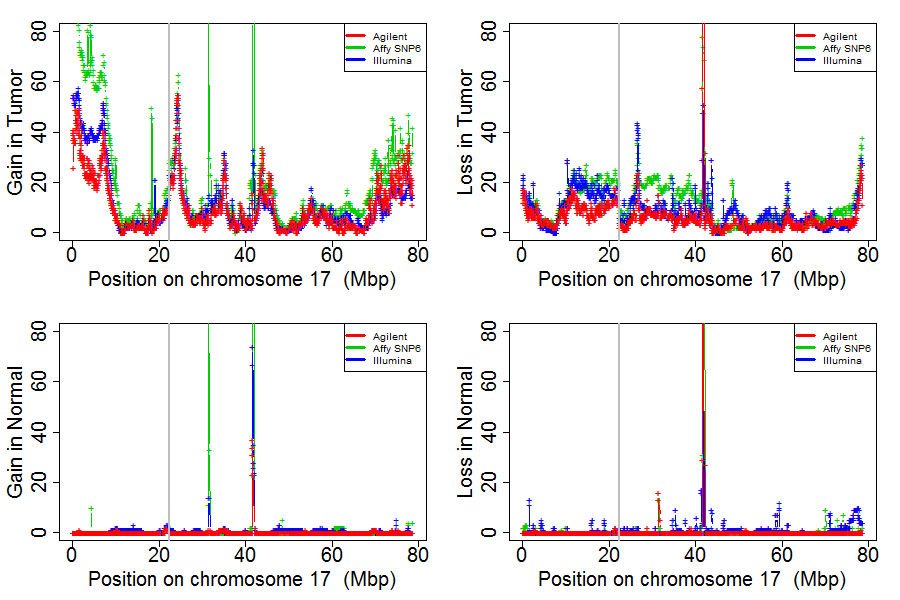


*Chromosome 18*


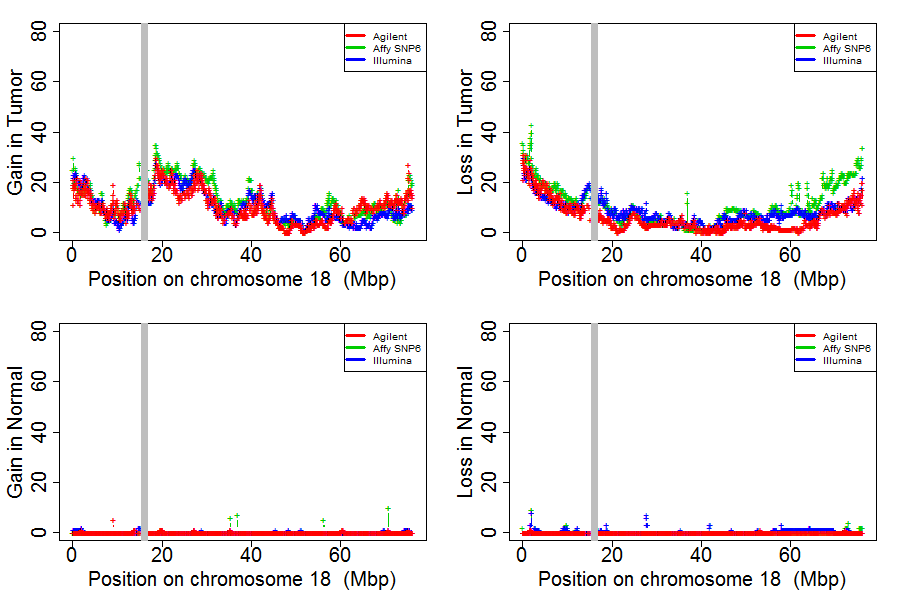


*Chromosome 19*


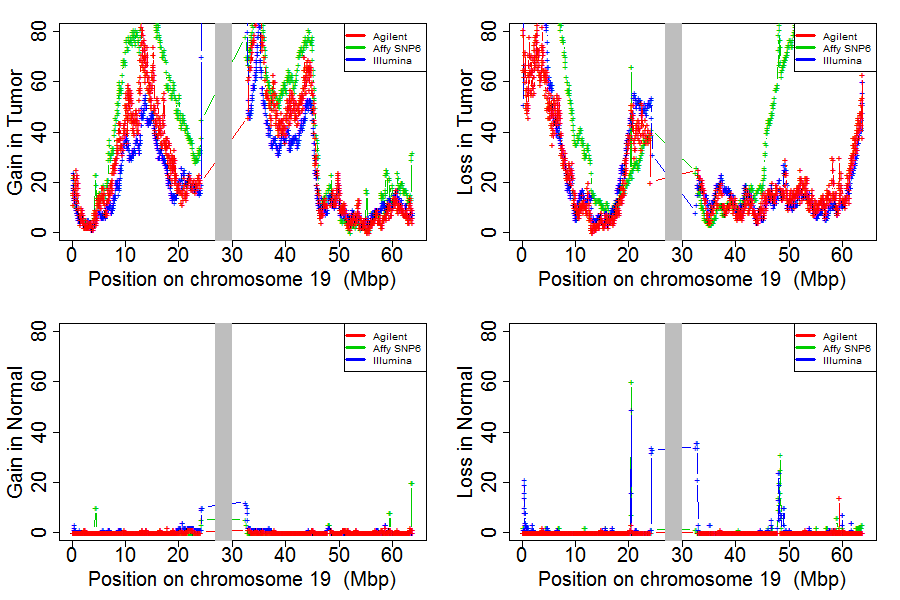


*Chromosome 20*


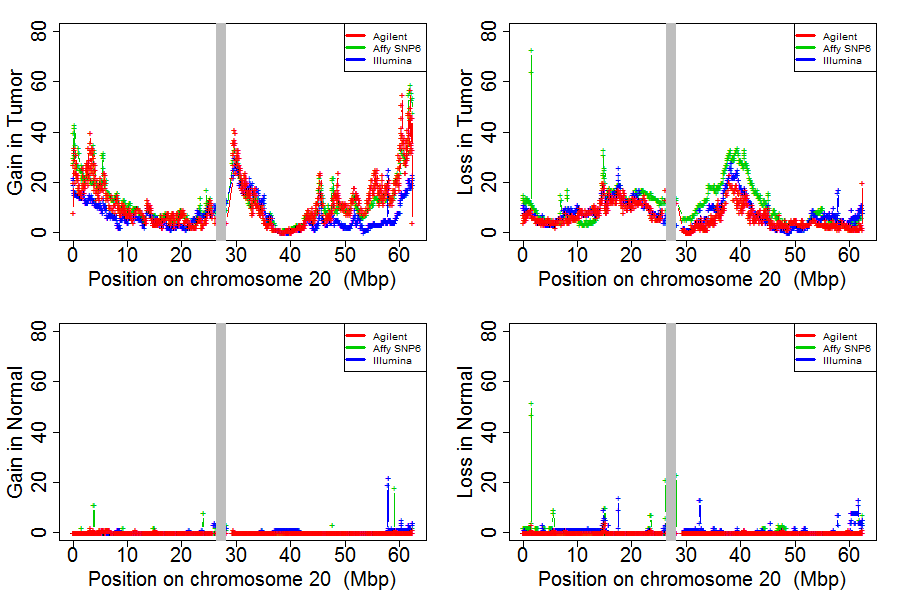


*Chromosome 21*


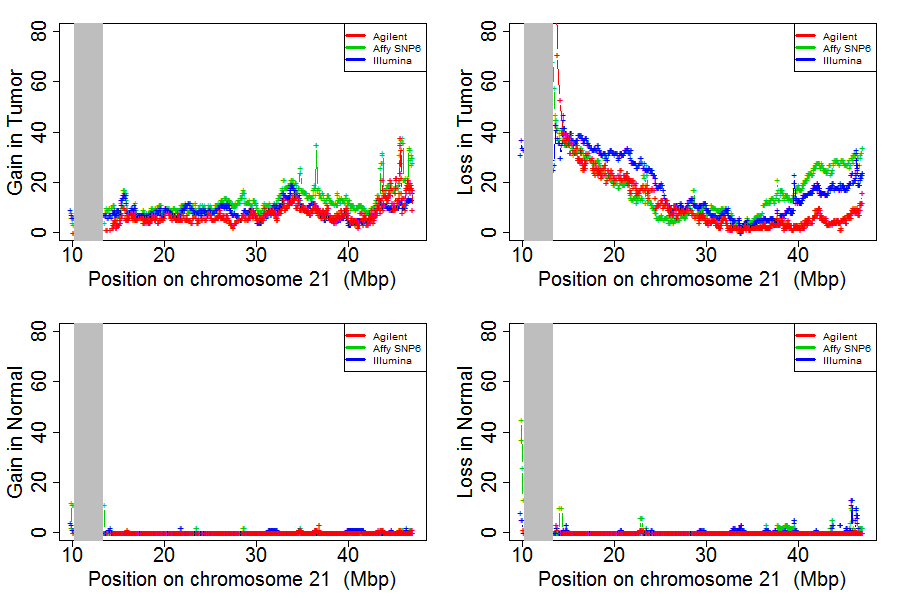


*Chromosome 22*


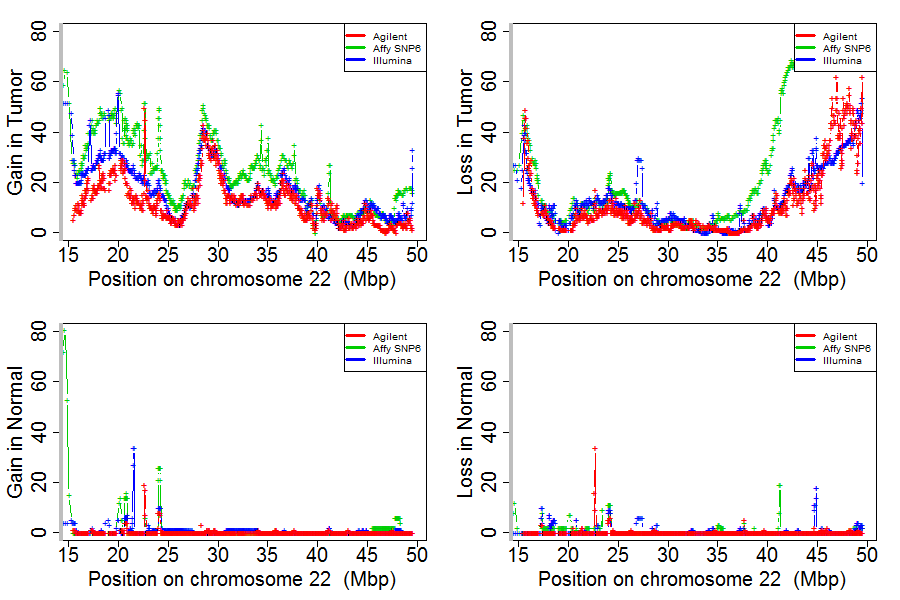


*Chromosome X*


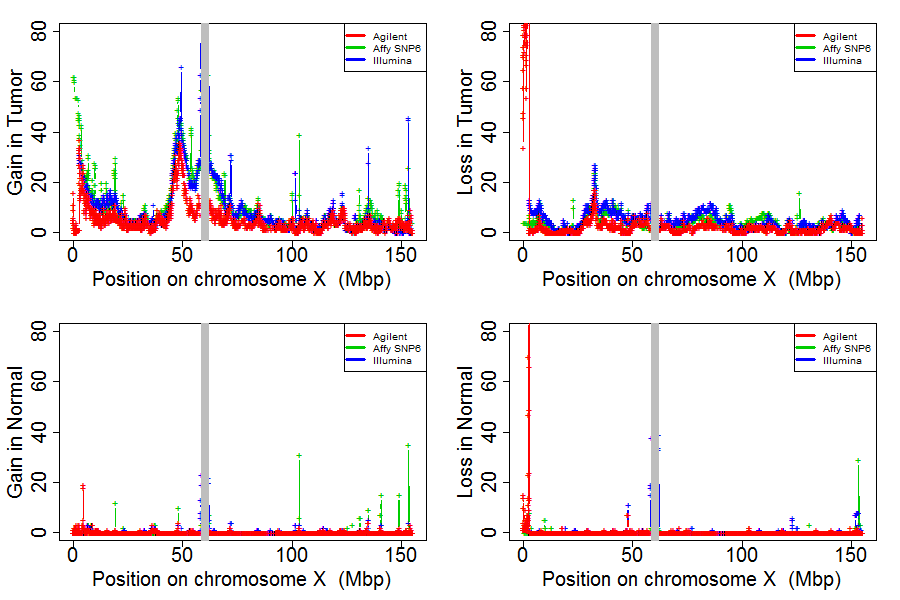


**Figure S2.1-S2.23.** Chromosome-wise frequency distributions of $f\mathrm{CNV}$ events in tumor (top) and normal (bottom) samples. Plots in red, green and blue represent data from Agilent, Affymetrix SNP6 and Illumina Platforms, respectively. $x$-axis represents physical position on a specific chromosome, while $y$-axis represents that of copy number gains or losses.

## Figure S3 Results of GISTIC analysis.

Figures S3.1-S3.2 showed the G-scores (top) and q-values (bottom) with respect to amplifications/deletions. The chromosomes (left) and cytobands (right) are arranged vertically from top to bottom, respectively. And different levels of significant thresholds are arranged from left to right. GISTIC version 2.0.12 [4] was used process probe level data of 587 samples obtained from Agilent arrays. Figures S3.3-S3.25 showed q-values on individual chromosomes by GISTIC 2.0 method. Lines in red and blue represent the q-values of focal gains and losses, respectively.

**
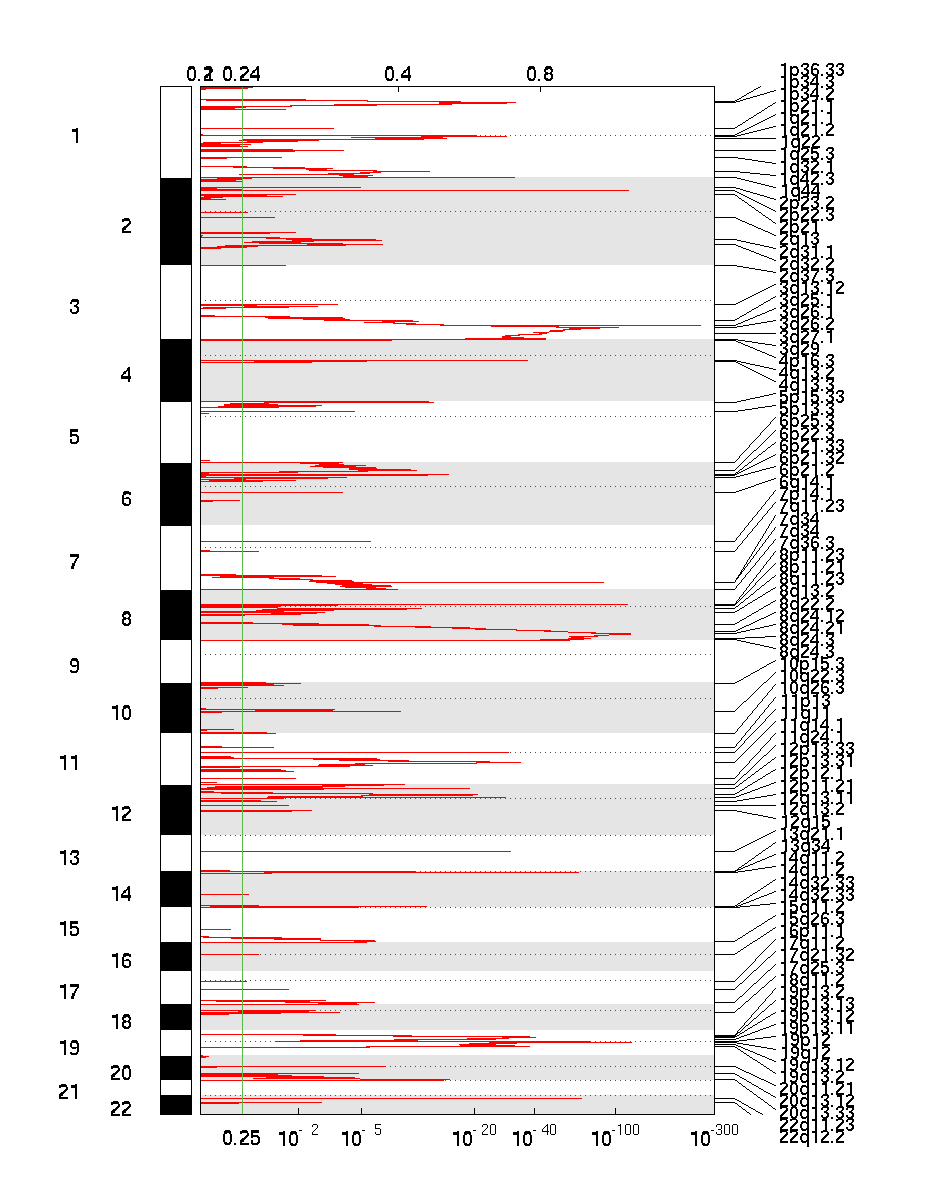
**

Q-value

G-Score

Chromosome

Cytoband

**Figure S3.1** The plot of the G-scores (top) and q-values (bottom) with respect to amplifications. The chromosomes (left) and cytobands (right) are arranged vertically from top to bottom, respectively. And different levels of significant thresholds are arranged from left to right. GISTIC version 2.0.12[4] was used process probe level data of 587 samples obtained from Agilent arrays.

**
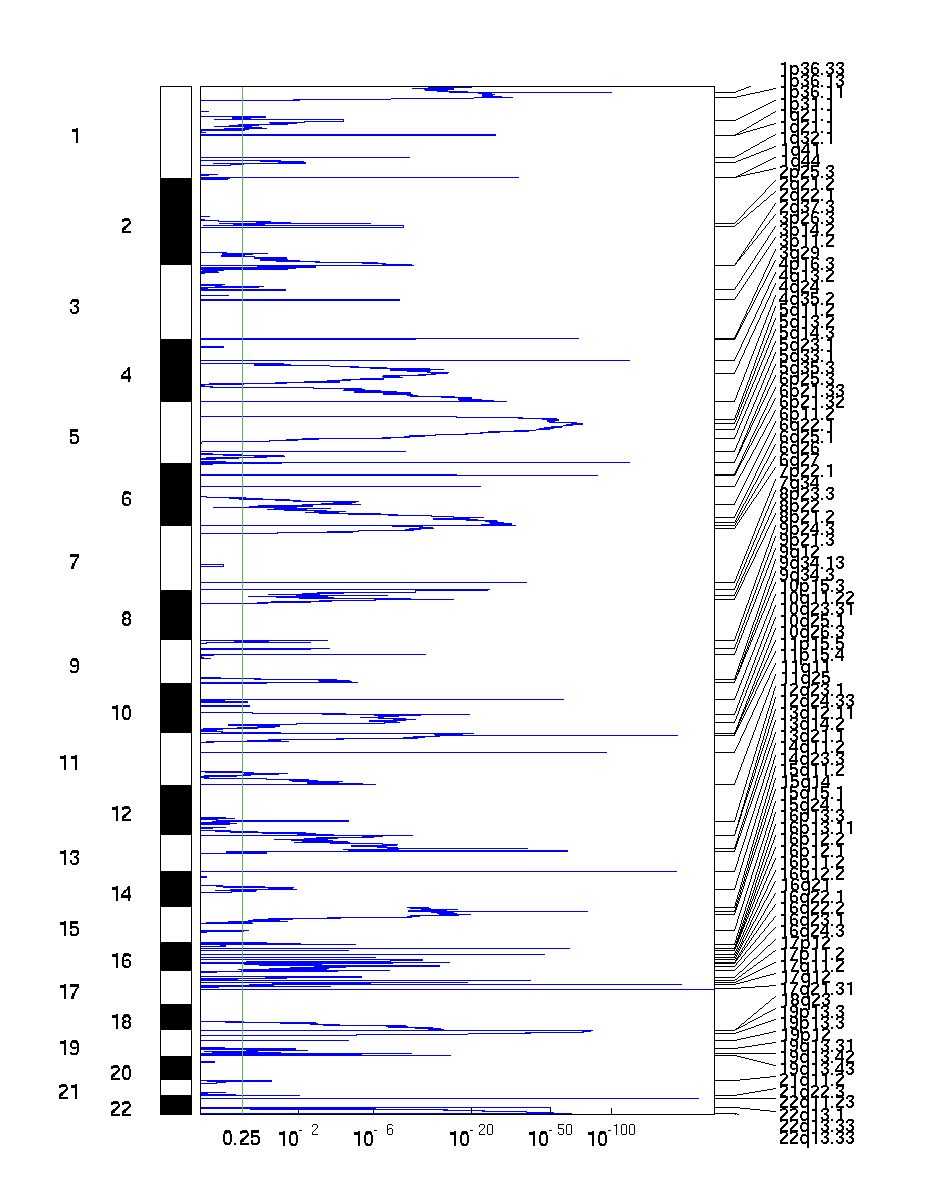
**

Q-value

G-Score

Cytoband

Chromosome

**Figure S3.2** The plot of the G-scores (top) and q-values (bottom) with respect to deletions. The chromosomes (left) and cytobands (right) are arranged vertically from top to bottom, respectively. And different levels of significant thresholds are arranged from left to right. GISTIC version 2.0.12[4] was used process probe level data of 587 samples obtained from Agilent arrays.

*Chromosome 1*

**
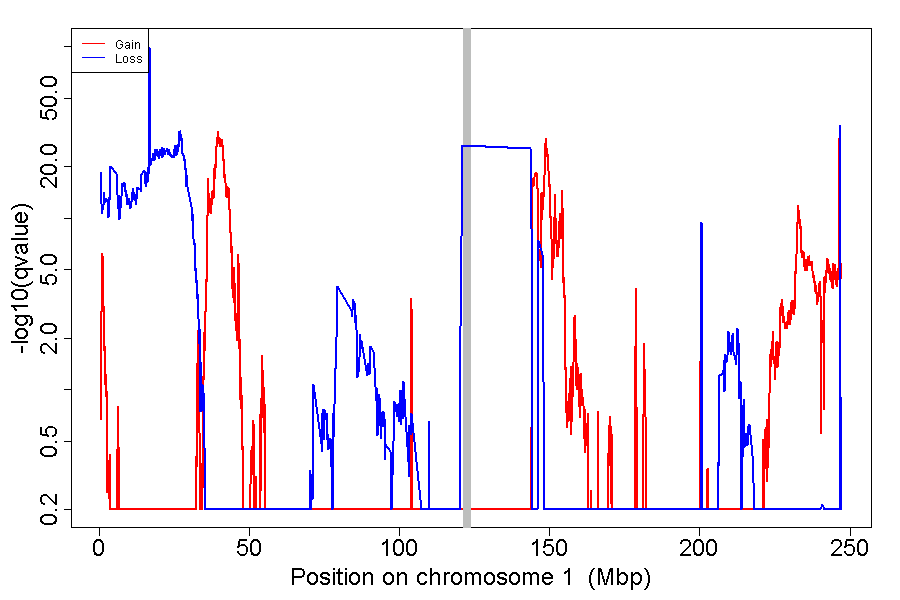
**

*Chromosome 2*

**
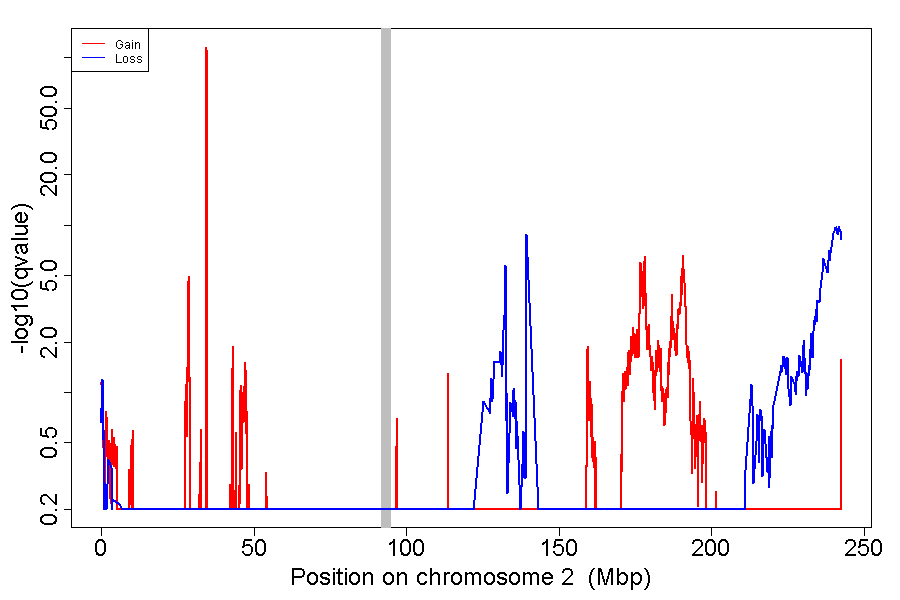
**

*Chromosome 3*

**
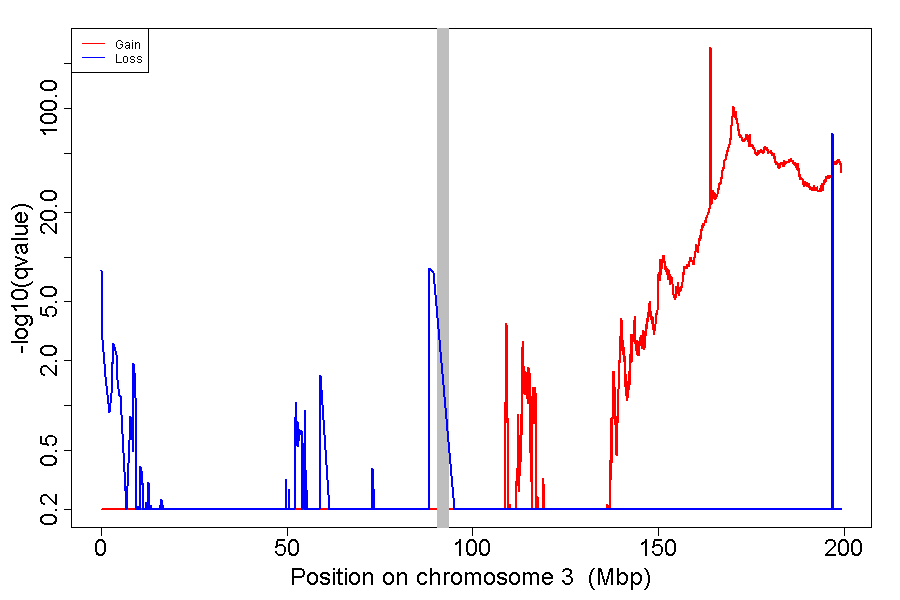
**

*Chromosome 4*

**
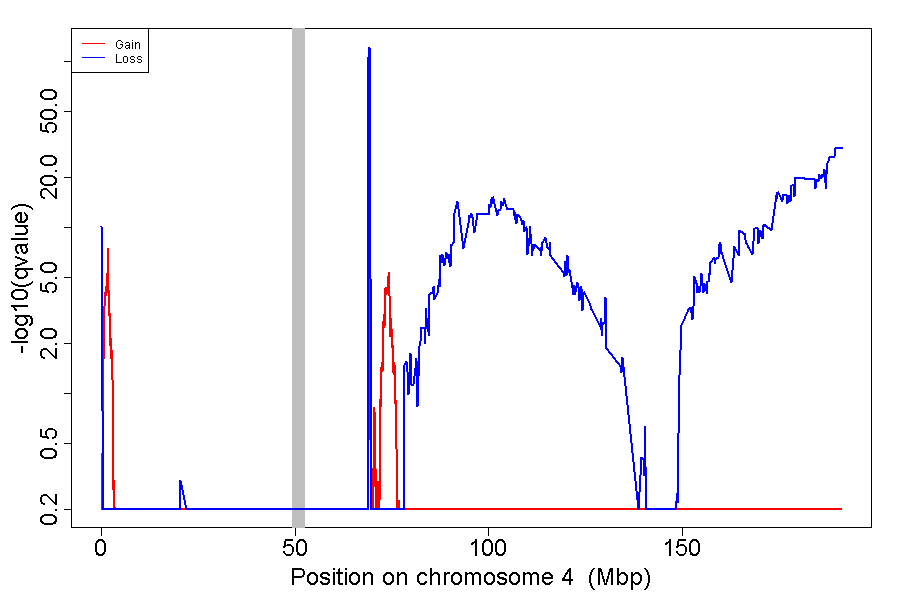
**

*Chromosome 5*

**
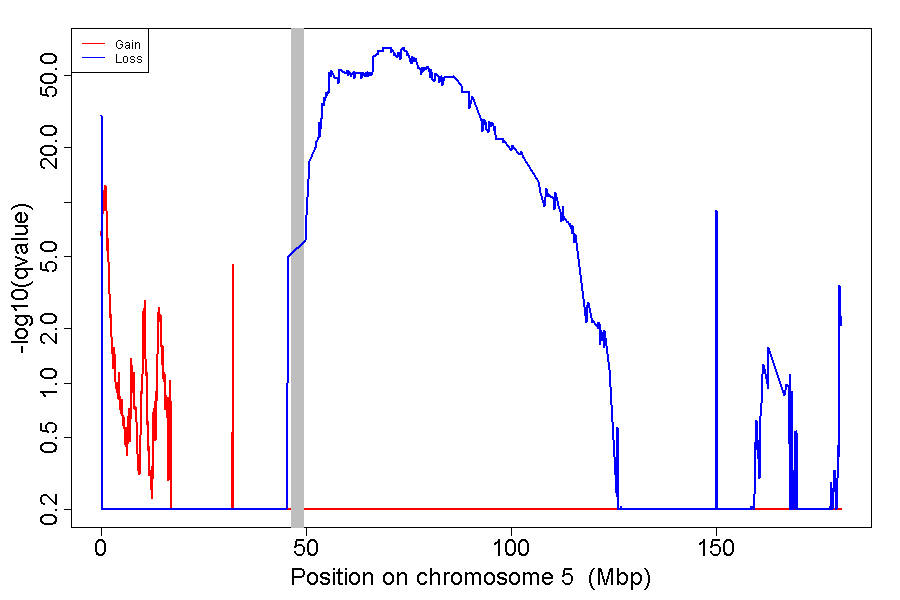
**

*Chromosome 6*

**
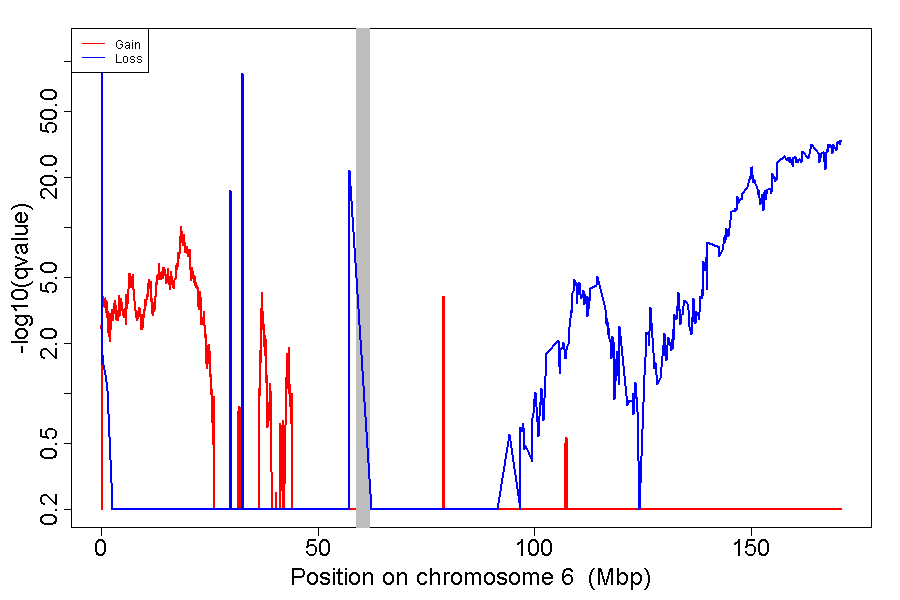
**

*Chromosome 7*

**
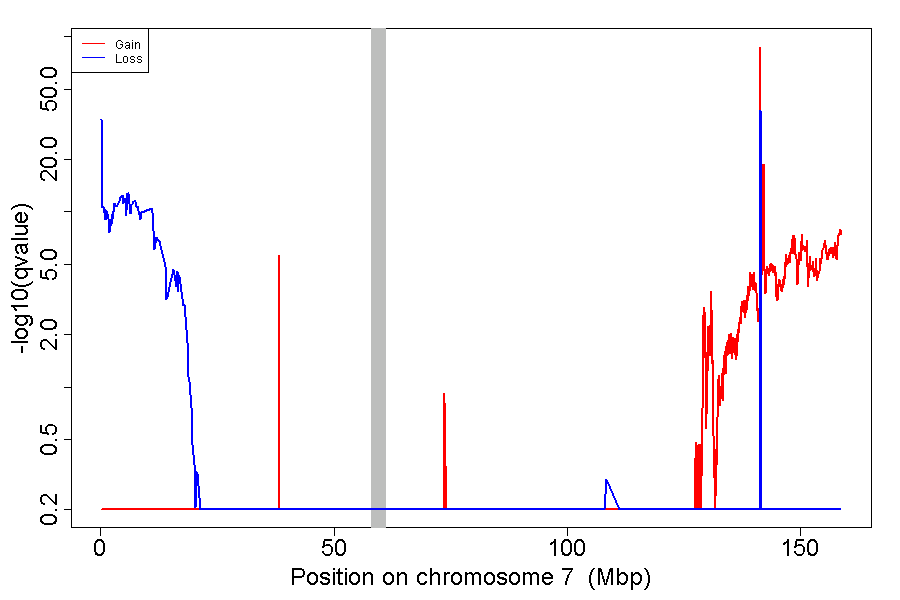
** *Chromosome 8*

**
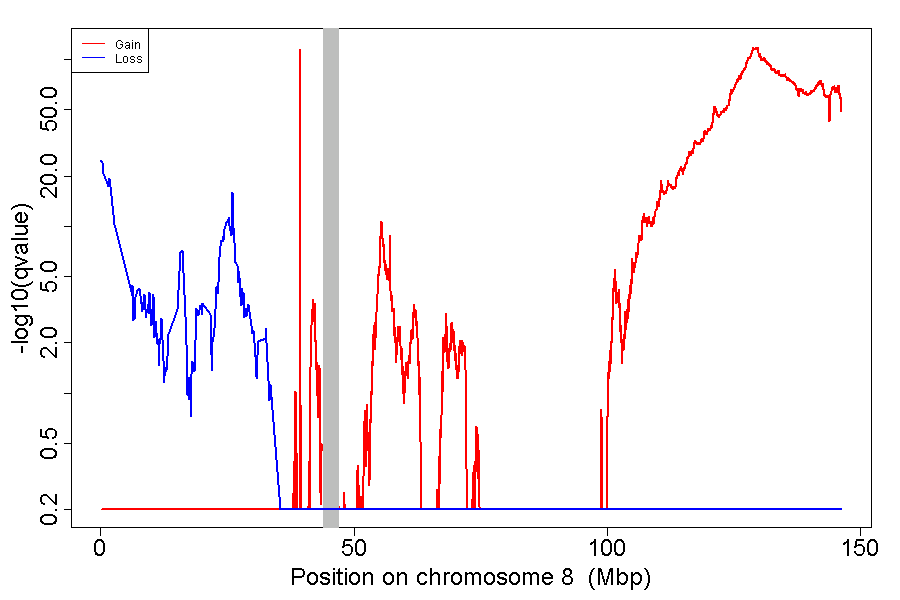
**

*Chromosome 9*

**
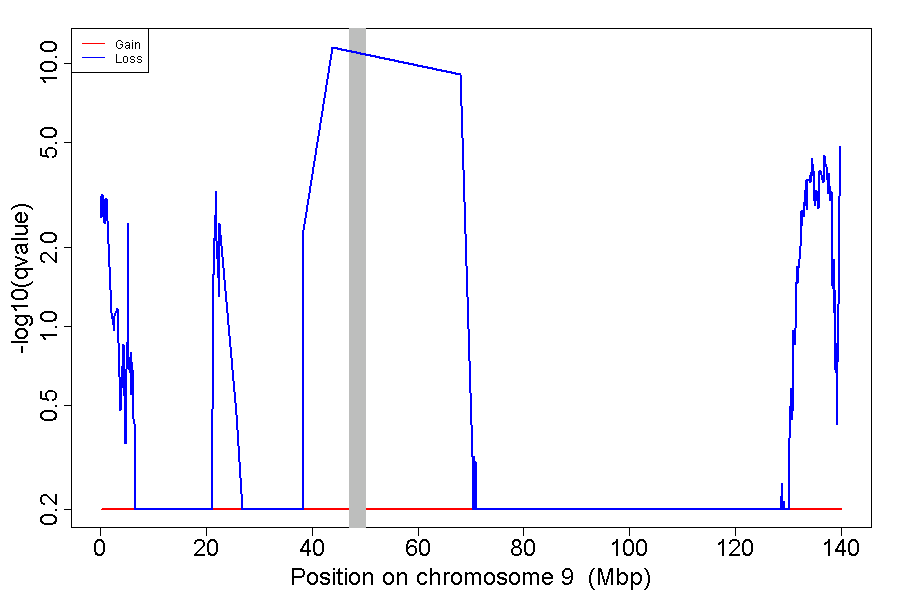
**

*Chromosome 10*

**
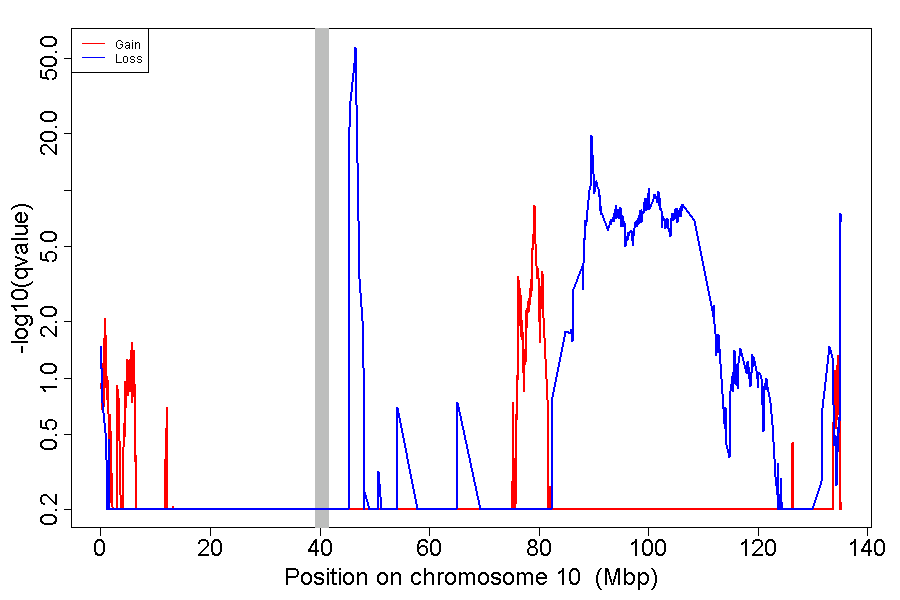
**

*Chromosome 11*

**
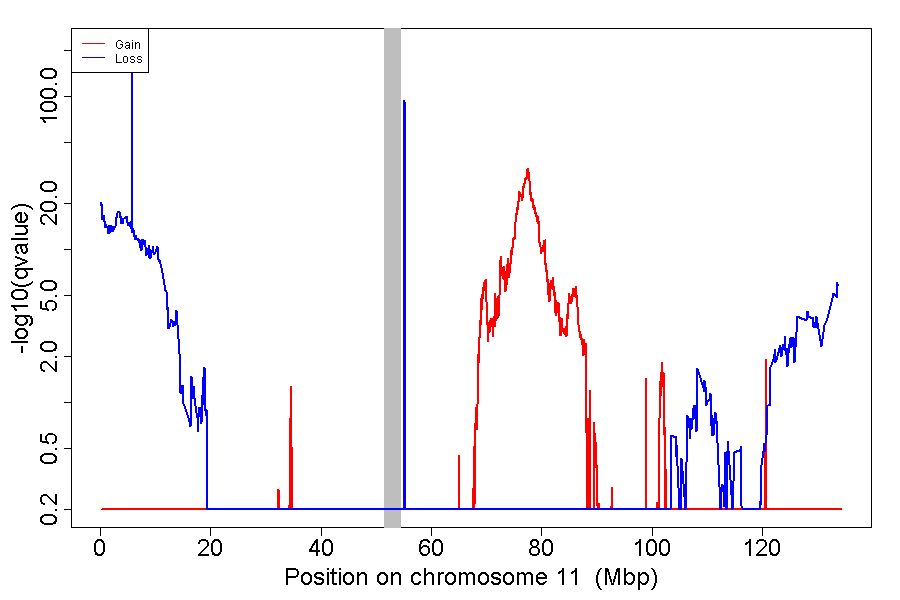
**

*Chromosome 12*

**
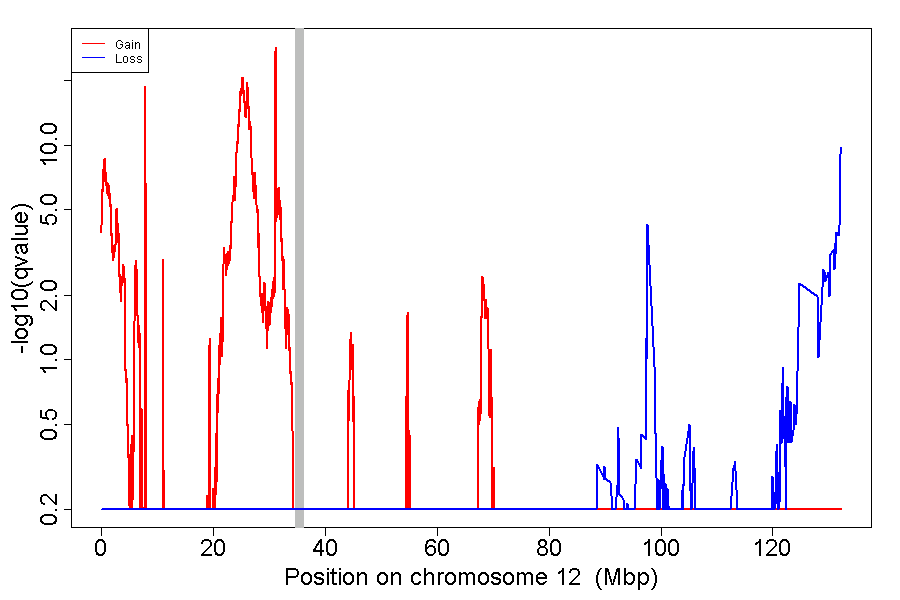
**

*Chromosome 13*

**
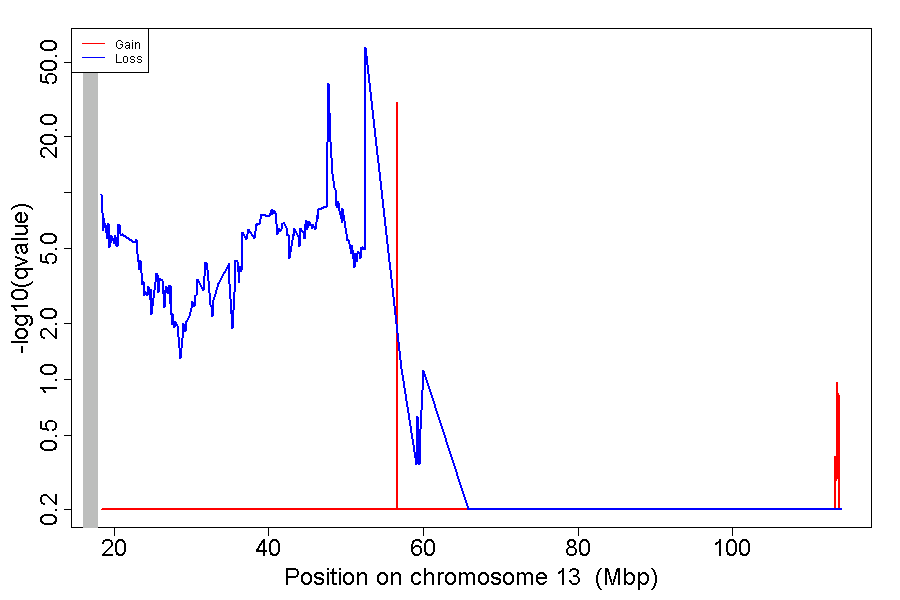
**

*Chromosome 14*

**
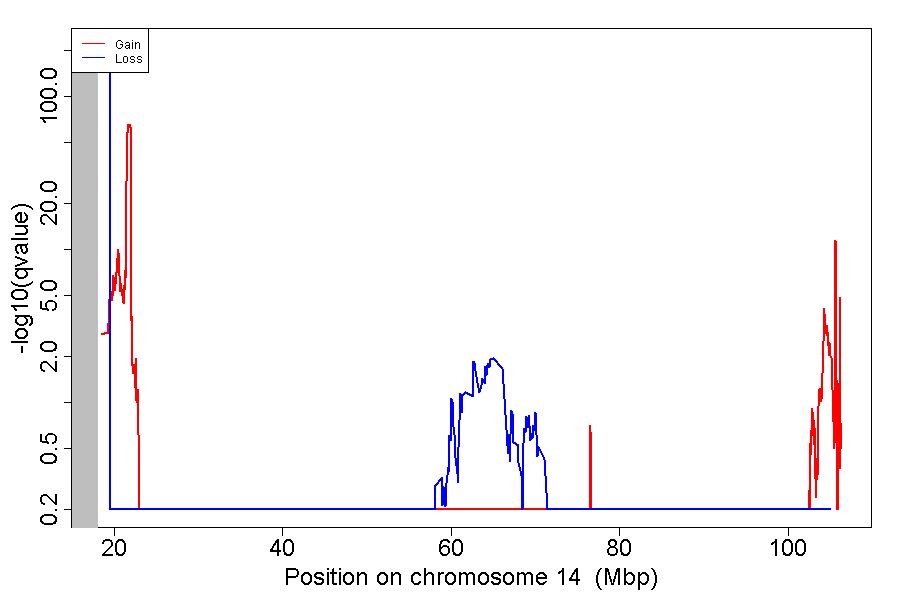
**

*Chromosome 15*

**
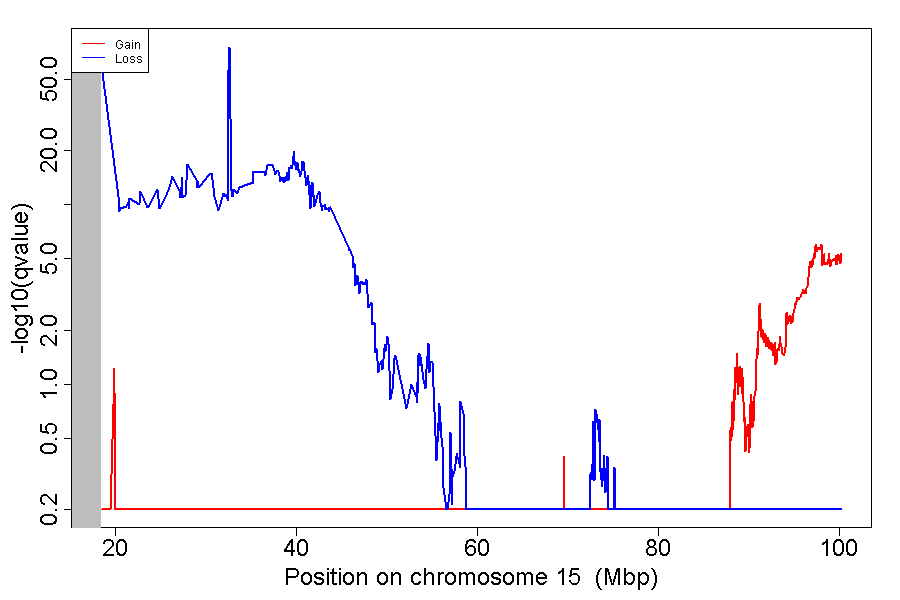
**

*Chromosome 16***
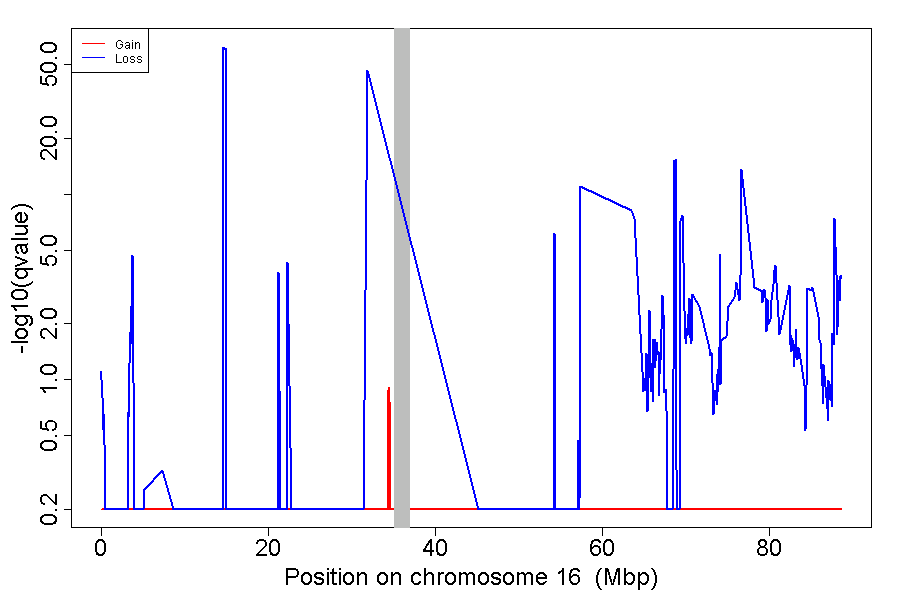
**

*Chromosome 17*

**
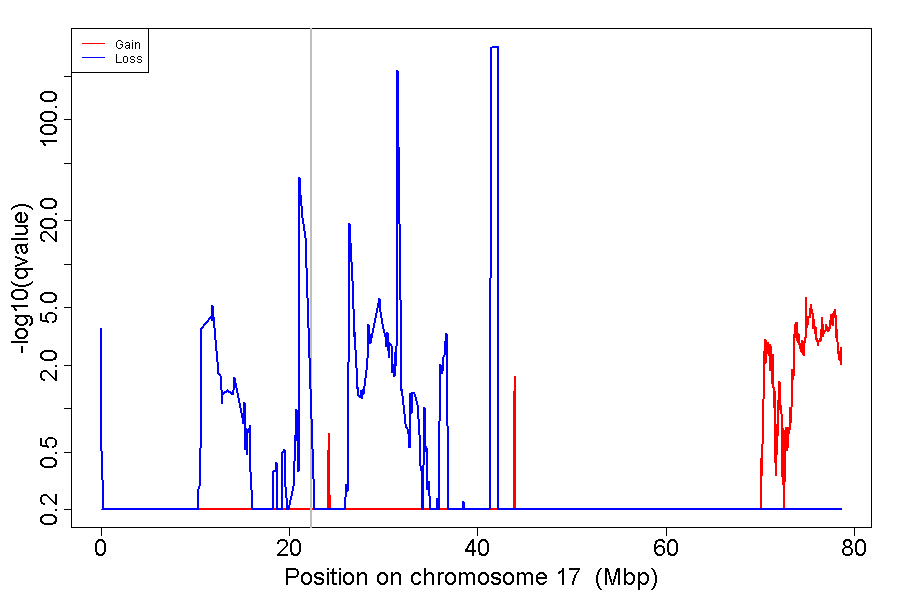
**

*Chromosome 18*

**
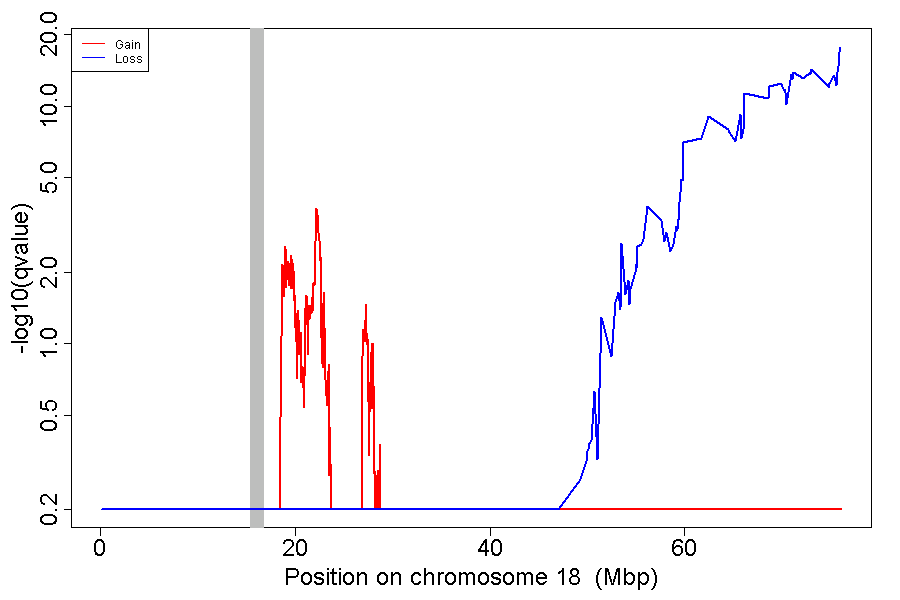
**

*Chromosome 19*

**
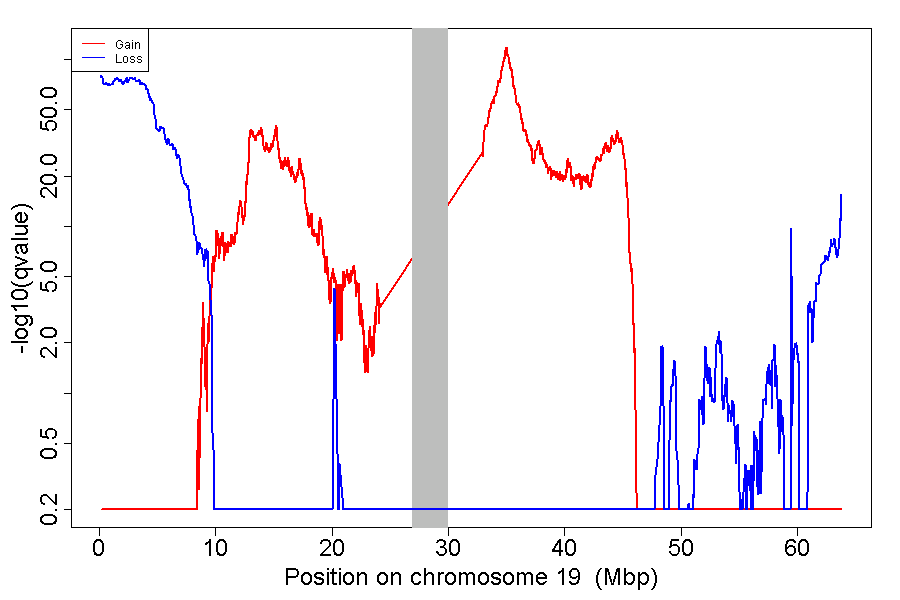
**

*Chromosome 20*

**
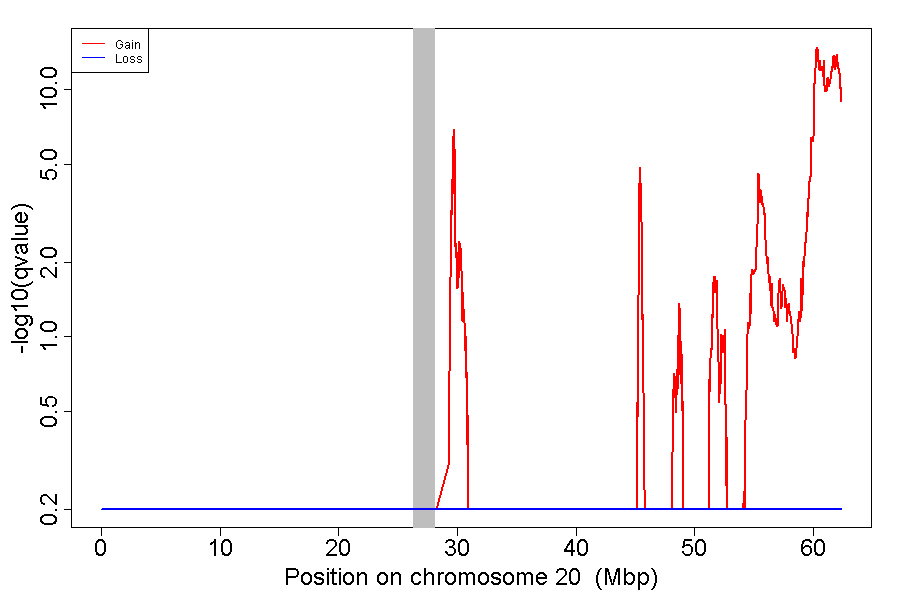
**

*Chromosome 21*

**
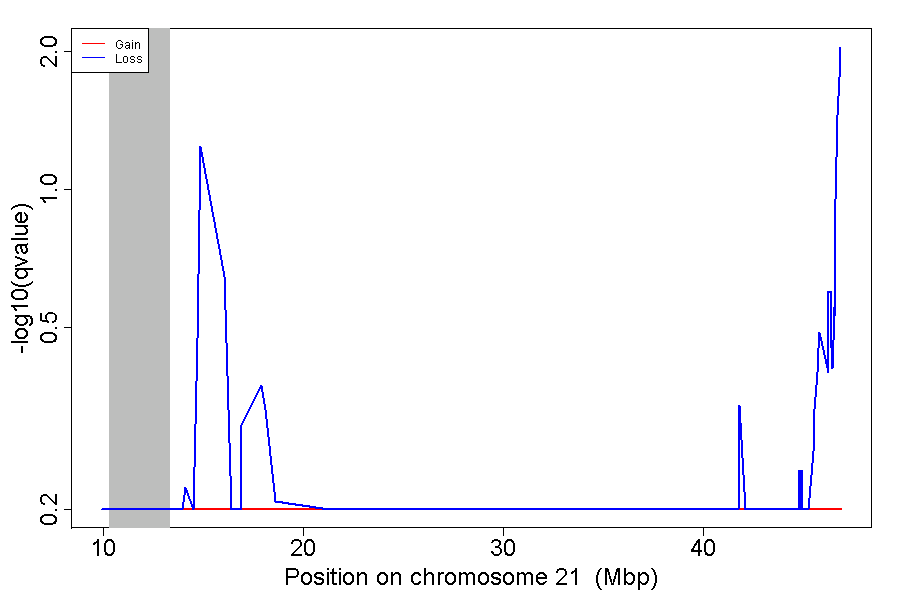
**

*Chromosome 22*

**
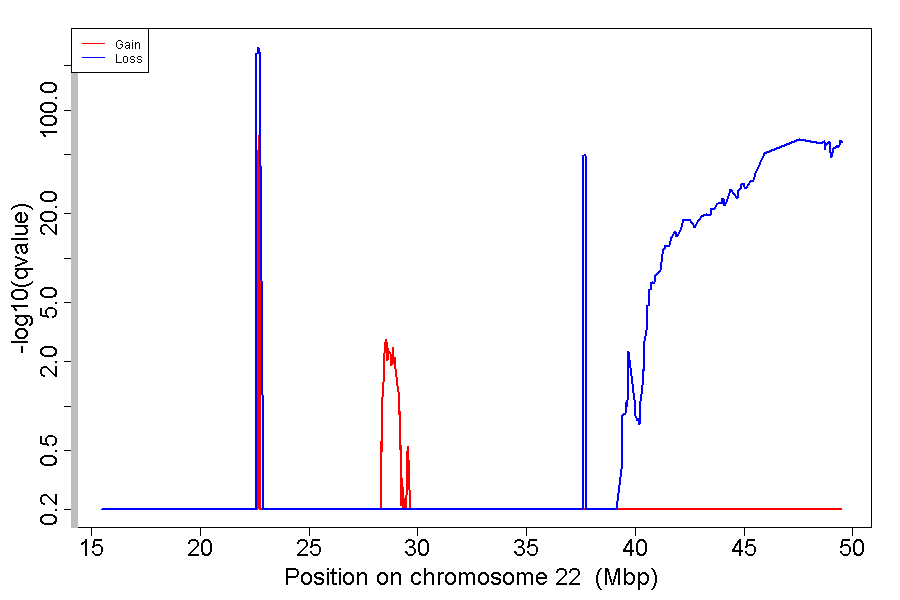
**

**Figure S3.3-S3.25.** Q-values on individual chromosomes by GISTIC 2.0 method. Lines in red and blue represent the q-values of focal gains and losses, respectively.

## Figure S4 Significance of association between CNV and PFS

**
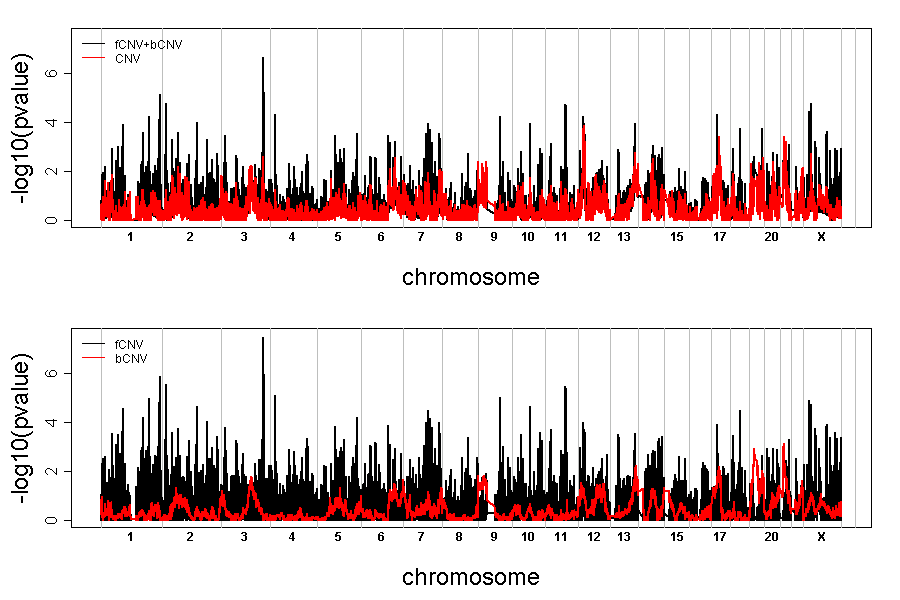
**

**Figure S4.1** Survival p value distributions throughout the whole genome (Platform: Agilent G4447A 1M). (A) $Surv\sim CNV$ *vs*. $Surv\sim fCNV+b\mathrm{CNV}$. P-values of CNV (in red) showed lower power than that of $f\mathrm{CNV}+b\mathrm{CNV}$ (in black) along the whole genome, suggesting that decomposition of the raw copy number data into focal and broad profiles will help improve the prediction power of prognostic markers in cancer progression. (B) $Surv\sim f\mathrm{CNV}$ *vs*. $Surv\sim b\mathrm{CNV}$ . P-values of $f\mathrm{CNV}$ (in black) had much more power than those of $b\mathrm{CNV}$ (in red) along the whole genome. This might imply that further decomposition of the full copy number profile into focal and broad profiles will help better understanding their roles in disease progression, respectively. $f\mathrm{CNVs}$ were found to have more clues in disease progression than broad ones.


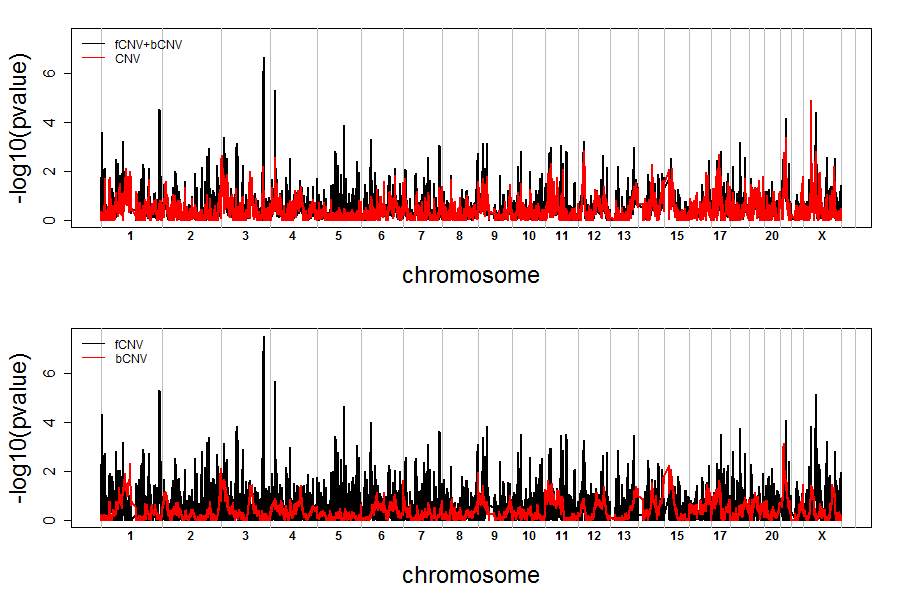


**Figure S4.2** Survival p value distributions throughout the whole genome (Platform: Affymetrix SNP 6.0). (A) $Surv\sim CNV$ *vs*. $Surv\sim fCNV+b\mathrm{CNV}$. P-values of CNV (in red) showed lower power than that of $f\mathrm{CNV}+b\mathrm{CNV}$ (in black) along the whole genome, suggesting that decomposition of the raw copy number data into focal and broad profiles will help improve the prediction power of prognostic markers in cancer progression. (B) $Surv\sim f\mathrm{CNV}$ *vs*. $Surv\sim b\mathrm{CNV}$ . P-values of $f\mathrm{CNV}$ (in black) had much more power than those of $b\mathrm{CNV}$ (in red) along the whole genome. This might imply that further decomposition of the full copy number profile into focal and broad profiles will help better understanding their roles in disease progression, respectively. $f\mathrm{CNVs}$ were found to have more clues in disease progression than broad ones.


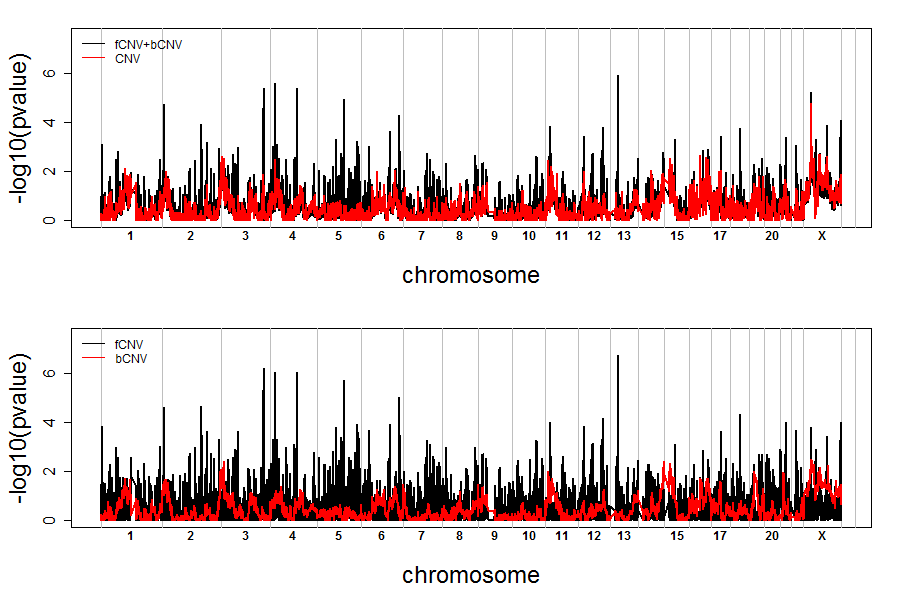


**Figure S4.3** Survival p-value distributions throughout the whole genome (Platform: Illumina Human 1MDuo). (A) $Surv\sim CNV$ *vs*. $Surv\sim fCNV+b\mathrm{CNV}$. P-values of CNV (in red) showed lower power than that of $f\mathrm{CNV}+b\mathrm{CNV}$ (in black) along the whole genome, suggesting that decomposition of the raw copy number data into focal and broad profiles will help improve the prediction power of prognostic markers in cancer progression. (B) $Surv\sim f\mathrm{CNV}$ *vs*. $Surv\sim b\mathrm{CNV}$ . P-values of $f\mathrm{CNV}$ (in black) had much more power than those of $b\mathrm{CNV}$ (in red) along the whole genome. This might imply that further decomposition of the full copy number profile into focal and broad profiles will help better understanding their roles in disease progression, respectively. $f\mathrm{CNVs}$ were found to have more clues in disease progression than broad ones.

## Figure S5 Significance of association between gene expression and PFS.


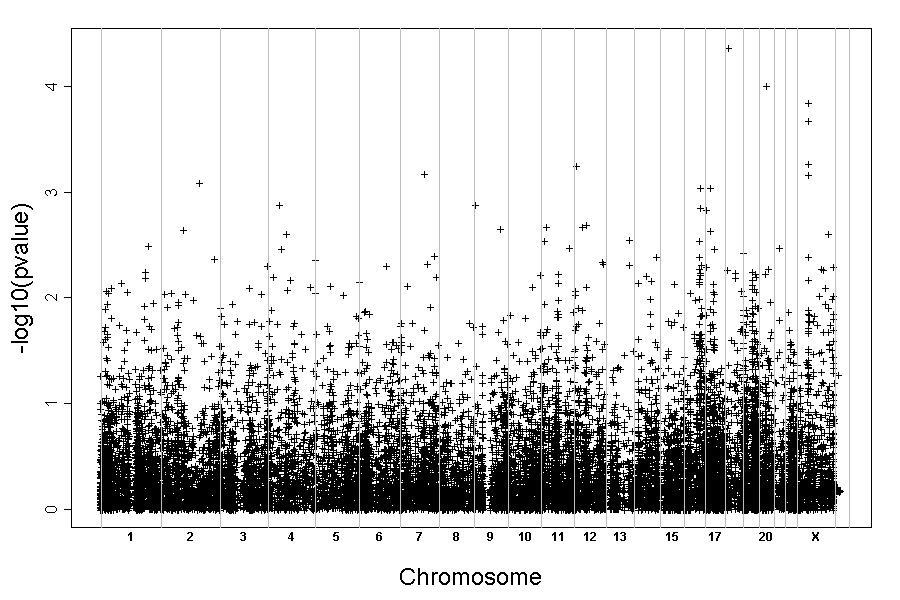
**Figure S5.1** Significance of association between gene expression and progression free survival time. The vertical axis showed the p-values obtained from single-variate Cox models relating gene expression values and progression free survival time in 301 samples. The gene expression values were obtained from Illumina RNAseq technology in terms of RPKM. The $x$-axis showed the chromosomal positions of the genes.


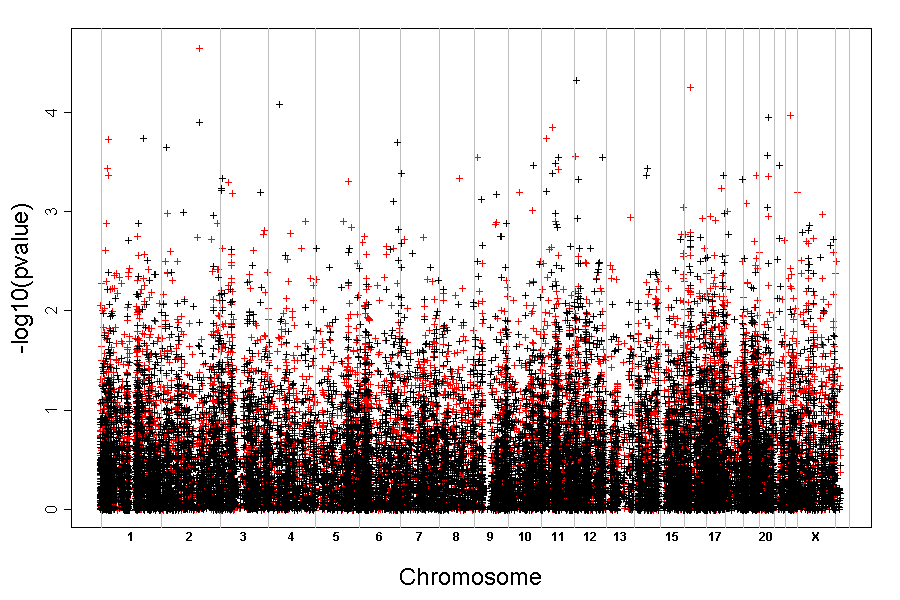


**Figure S5.2** Significance of association between gene expression and progression free survival time. The $x$-axis showed the chromosomal positions of the genes. The vertical axis showed the p-values obtained from single-variate Cox models relating gene expression values and progression free survival time. The gene expression values were obtained from Agilent G4502A arrays (red points) and Affymetrix U133A arrays (black points). There are 569 samples obtained from Affymetrix arrays and 562 samples from Agilent arrays.

## Figure S6 Simulation results of the sensitivity and specificity for our decomposition method


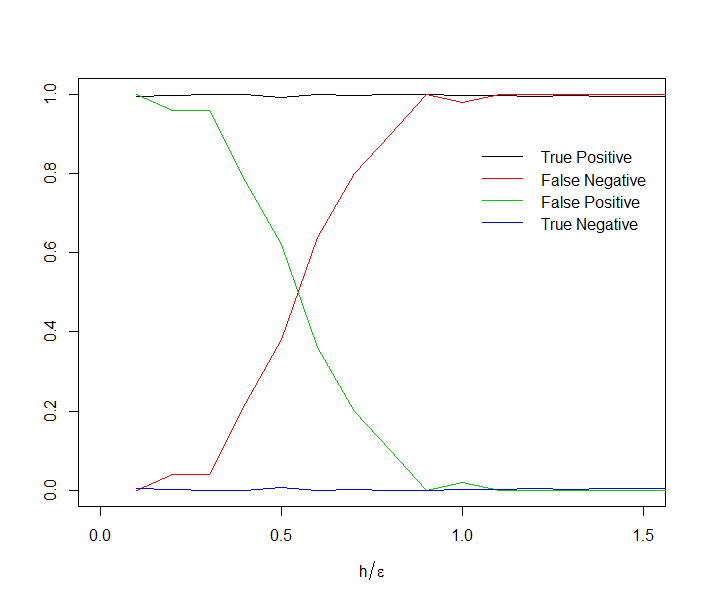


**Figure S6.1. Simulation results of the sensitivity and specificity for our decomposition method with respect to signal/noise level.**


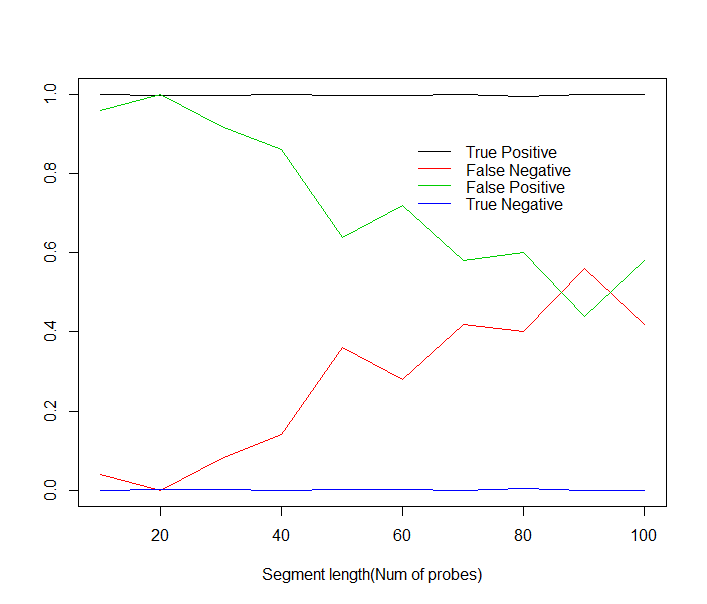
 **Figure S6.2. Simulation results of the sensitivity and specificity for our decomposition method with respect to segmentation length(number of probes).**


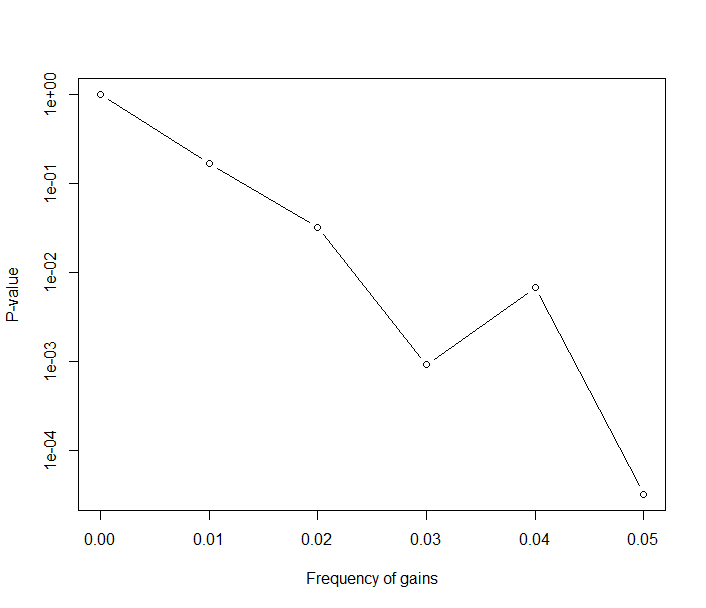


**Figure S6.3. Simulation results of ratio test significance (p-value) for our decomposition method with respect to the levels of driver frequencies.**

## Table S1 Sample information

Table S1 showed the list of patients ids included in our analysis. It contained the genomic data platform, tissue type (primary tumor or adjacent normal tissue) and clinical outcome (progression free survival (PFS) time and censoring status). There are three different platforms including the Agilent G4447A (short for "G4447A"), Affymetrix SNP6 ("SNP6") and the Illumina 1M-Duo ("1M-Duo"). And the columns are as follows:

- "Sample.ID", TCGA patient id
- "tumor.CNV.G4447A": “Yes” represents the current sample had the copy number data of tumor tissues tested on the Agilent G4447A platform; “No” represents the current sample didn’t the copy number data of tumor tissues tested on the Agilent G4447A platform.
- "tumor.CNV.SNP6": “Yes” represents the current sample had the copy number data of tumor tissues tested on the Affymetrix SNP6 platform; “No” represents the current sample didn’t the copy number data of tumor tissues tested on the Affymetrix SNP6 platform.
- "tumor.CNV.1M-Duo": “Yes” represents the current sample had the copy number data of tumor tissues tested on the Illumina 1M-Duo platform; “No” represents the current sample didn’t the copy number data of tumor tissues tested on the Illumina 1M-Duo platform.
- "tumor.EXP.Illumina": “Yes” represents the current sample had the mRNA expression data of tumor tissues tested on the Illumina RNAseq platform; “No” represents the current sample didn’t the mRNA expression of tumor tissues tested on the Illumina RNAseq platform.
- "tumor.EXP.U133A": “Yes” represents the current sample had the mRNA expression data of tumor tissues tested on the Affymetrix U133A platform; “No” represents the current sample didn’t the mRNA expression of tumor tissues tested on the Affymetrix U133A platform.
- "tumor.EXP.244K": “Yes” represents the current sample had the mRNA expression data of tumor tissues tested on the Agilent 244k platform; “No” represents the current sample didn’t the mRNA expression of tumor tissues tested on the Agilent 244k platform.
- "normal.CNV.G4447A": “Yes” represents the current sample had the copy number data of normal tissues tested on the Agilent G4447A platform; “No” represents the current sample didn’t the copy number data of normal tissues tested on the Agilent G4447A platform.
- "normal.CNV.SNP6": “Yes” represents the current sample had the copy number data of normal tissues tested on the Affymetrix SNP6 platform; “No” represents the current sample didn’t the copy number data of normal tissues tested on the Affymetrix SNP6 platform.
- "normal.CNV.1M-Duo": “Yes” represents the current sample had the copy number data of normal tissues tested on the Illumina 1M-Duo platform; “No” represents the current sample didn’t the copy number data of normal tissues tested on the Illumina 1M-Duo platform.
- "survival_time" and “survival_status": represents the censored PFS time and status in days for the current sample.
- "days_to_new_tumor_event_after_initial_treatment","days_to_death", “days_to_tumor_progression","days_to_tumor_recurrence","days_to_last_followup" represent the number of days that the current sample diagnosed to have a recurrent tumor event, new tumor event after initial treatment, tumor progression, recurrence or last known contact if the patient was alive.

## Table S2 Distributions of focal gains/losses

Table S2 showed the distributions of focal gains and losses. There are three different platforms including the Agilent G4447A (short for "G4447A"), Affymetrix SNP6 ("SNP6") and the Illumina 1M-Duo ("1M-Duo"). And the columns are as follows:

- Chromosome
- Physical location (Genome version: hg18)
- "focal.loss.95%CI.G4447A" represents the confidence intervals for focal losses on the Agilent G4447A platform.
- "focal.gain.95%CI.G4447A" represents the confidence intervals for focal gains on the Agilent G4447A platform.
- "loss.peak.width.G4447A" represents the peak width of the focal loss regions identified on the Agilent G4447A platform.
- "gain.peak.width.G4447A" represents the peak width of the focal gain regions identified on the Agilent G4447A platform.
- "is.peak.focal.loss.G4447A" represents whether there is a peak position in terms of the focal loss.
- "is.peak.focal.gain.G4447A" represents whether there is a peak position in terms of the focal gain.
- "Tumor.focal.loss.G4447A" represents the frequency of focal losses across tumor samples on the Agilent G4447A platform.
- "Tumor.focal.gain.G4447A" represents the frequency of focal gains across tumor samples on the Agilent G4447A platform.
- "Tumor.broad.loss.G4447A" represents the frequency of broad losses across tumor samples on the Agilent G4447A platform.
- "Tumor.broad.gain.G4447A" represents the frequency of broad gains across tumor samples on the Agilent G4447A platform.
- "Normal.focal.loss.G4447A" represents the frequency of focal losses across normal samples on the Agilent G4447A platform.
- "Normal.focal.gain.G4447A" represents the frequency of focal gains across normal samples on the Agilent G4447A platform.
- "Normal.broad.loss.G4447A" represents the frequency of broad losses across normal samples on the Agilent G4447A platform.
- "Normal.broad.gain.G4447A" represents the frequency of focal gains across normal samples on the Agilent G4447A platform.
- "is.CNP.G4447A" represents whether the current probe site is a CNP in terms of the Agilent G4447A platform.
- "Ratio.test.pvalue.G4447A" represents whether the number of focal gains is significantly different from the number of losses on the Agilent G4447A platform.
- "Tumor.focal.loss.SNP6" represents the frequency of focal losses across tumor samples on the Affymetrix SNP6 platform.
- "Tumor.focal.gain.SNP6" represents the frequency of focal gains across tumor samples on the Affymetrix SNP6 platform.
- "Tumor.broad.loss.SNP6" represents the frequency of broad losses across tumor samples on the Affymetrix SNP6 platform.
- "Tumor.broad.gain.SNP6" represents the frequency of broad gains across tumor samples on the Affymetrix SNP6 platform.
- "Normal.focal.loss.SNP6" represents the frequency of focal losses across normal samples on the Affymetrix SNP6 platform.
- "Normal.focal.gain.SNP6" represents the frequency of focal gains across normal samples on the Affymetrix SNP6 platform.
- "Normal.broad.loss.SNP6" represents the frequency of broad losses across normal samples on the Affymetrix SNP6 platform.
- "Normal.broad.gain.SNP6" represents the frequency of focal gains across normal samples on the Affymetrix SNP6 platform.
- "is.CNP.SNP6" represents whether the current probe site is a CNP in terms of the Affymetrix SNP6 platform.
- "Ratio.test.pvalue.SNP6" represents whether the number of focal gains is significantly different from the number of losses on the Affymetrix SNP6 platform.
- "Tumor.focal.loss.1M-Duo" represents the frequency of focal losses across tumor samples on the Illumina 1M-Duo platform.
- "Tumor.focal.gain.1M-Duo" represents the frequency of focal gains across tumor samples on the Illumina 1M-Duo platform.
- "Tumor.broad.loss.1M-Duo" represents the frequency of broad losses across tumor samples on the Illumina 1M-Duo platform.
- "Tumor.broad.gain.1M-Duo" represents the frequency of broad gains across tumor samples on the Illumina 1M-Duo platform.
- "Normal.focal.loss.1M-Duo" represents the frequency of focal losses across normal samples on the Illumina 1M-Duo platform.
- "Normal.focal.gain.1M-Duo" represents the frequency of focal gains across normal samples on the Illumina 1M-Duo platform.
- "Normal.broad.loss.1M-Duo" represents the frequency of broad losses across normal samples on the Illumina 1M-Duo platform.
- "Normal.broad.gain.1M-Duo" represents the frequency of focal gains across normal samples on the Illumina 1M-Duo platform.
- "is.CNP.1M-Duo" represents whether the current probe site is a CNP in terms of the Illumina 1M-Duo platform.
- "Ratio.test.pvalue.1M-Duo" represents whether the number of focal gains is significantly different from the number of losses on the Illumina 1M-Duo platform.

## Table S3 List of genes encoded in the recurrent focal event regions.

Table S3 showed the regions identified using the following criteria: (1) Peak height >=8, *i.e*., no less than 8 patients have the focal gains (losses) at the peak position; (2) The 95% CI of the peak position is less than 1 million base pairs; (3) Less than 4 gains or losses were found in the normal tissue samples within the 95% CI; (4) The number of focal gains must be significantly different from the number of losses in the same region.

- Type: ‘Amp/Del’ represents current focal region is a gain or loss region.
- Chromosome
- Physical position (Genome version: hg18)
- CI(Mb): the 95% confidence interval of the focal peak positions.
- “Known cancer driver genes” represents the identified cancer drivers. The up-arrow ^↑^ represents the current gene is a known oncogene; the down arrow ^↓^ represents the current gene is a known TSGs; and ^↕^  represents the gene that plays dual roles both as an oncogene and a TSG.

## Table S4 Prognostic results of ovarian cancer progression using fCNV on three platforms.

Table S4 showed the p-values of the cox model between CNV and PFS. And the columns are as follows:

- Chromosome
- Physical position (Genome version: hg18)
- "focal.coef.G4447A" represents the estimated coefficient (log-hazard ratio) of focal copy number profiles using the $M_{f,bcnv}$ model to infer the association of clinical outcomes with the copy number data of the Agilent G4447A platform.
- "broad.coef.G4447A" represents the estimated coefficient (log-hazard ratio) of broad copy number profiles using the $M_{f,bcnv}$ model to infer the association of clinical outcomes with the copy number data of the Agilent G4447A platform.
- "focal.pvalue.G4447A" represents the estimated significance of focal copy number profiles using the $M_{f,bcnv}$ model to infer the association of clinical outcomes with the copy number data of the Agilent G4447A platform.
- "broad.pvalue.G4447A" represents the estimated significance of broad copy number profiles using the $M_{f,bcnv}$ model to infer the association of clinical outcomes with the copy number data of the Agilent G4447A platform.
- "wald.test.pvalue.G4447A" represents the estimated *wald* test pvalue of the $M_{f,bcnv}$ model to infer the association of clinical outcomes with the copy number data of the Agilent G4447A platform.
- "focal.pvalue.SNP6" represents the estimated significance of focal copy number profiles using the $M_{f,bcnv}$ model to infer the association of clinical outcomes with the copy number data of the Affymetrix SNP6 platform.
- "broad.pvalue.SNP6" represents the estimated significance of broad copy number profiles using the $M_{f,bcnv}$ model to infer the association of clinical outcomes with the copy number data of the Affymetrix SNP6 platform.
- "wald.test.pvalue.SNP6" represents the estimated *wald* test pvalue of the $M_{f,bcnv}$ model to infer the association of clinical outcomes with the copy number data of the Affymetrix SNP6 platform.
- "focal.pvalue.1M-Duo" represents the estimated significance of focal copy number profiles using the $M_{f,bcnv}$ model to infer the association of clinical outcomes with the copy number data of the Illumina 1M-Duo platform.
- "broad.pvalue.1M-Duo" represents the estimated significance of broad copy number profiles using the $M_{f,bcnv}$ model to infer the association of clinical outcomes with the copy number data of the Illumina 1M-Duo platform.
- "wald.test.pvalue.1M-Duo" represents the estimated *wald* test pvalue of the $M_{f,bcnv}$ model to infer the association of clinical outcomes with the copy number data of the Illumina 1M-Duo platform.

# Abbreviations

Affymetrix SNP 6.0 Affymetrix Genome-Wide Human SNP Array 6.0

Agilent G4447A 1M Agilent SurePrint G3 Human CGH Microarray Kit 1x1M

Agilent 244K Agilent 244K Custom Human Gene Expression G4502A-07-3

bCNV broad Copy Number Variation

CI Confidence Interval

CNP Copy Number Polymorphism

fCNV focal Copy Number Variation

GISTIC Genomic Identification of Significant Targets In Cancer

Illumima 1M-Duo Illumina Human1M-Duo BeadChip

Mb Million base pair

RPKM Reads Per Kilobase per Million of mapped reads

RNAseq Whole Transcriptome Shotgun Sequencing

SNP Single Nucleotide Polymorphism

TCGA The Cancer Genome Atlas

PFS Progression Free Survival

TSG Tumor Suppressor Gene

Affymetrix U133A Affymetrix Human Genome HTS U133A 2.0
